# Orientin Prolongs the Longevity of *Caenorhabditis elegans* and Postpones the Development of Neurodegenerative Diseases via Nutrition Sensing and Cellular Protective Pathways

**DOI:** 10.1155/2022/8878923

**Published:** 2022-02-21

**Authors:** Yuan Qu, Lin Shi, Yu Liu, Lv Huang, Huai-Rong Luo, Gui-Sheng Wu

**Affiliations:** ^1^Key Laboratory for Aging and Regenerative Medicine, Department of Pharmacology School of Pharmacy, Southwest Medical University, Luzhou, Sichuan 646000, China; ^2^Department of Pharmacy Children's Hospital of Chongqing Medical University, National Clinical Research Center for Child Health and Disorders, Ministry of Education Key Laboratory of Child Development and Disorders, Chongqing Key Laboratory of Pediatrics, Chongqing 400000, China; ^3^Central Nervous System Drug Key Laboratory of Sichuan Province, Luzhou, Sichuan 646000, China; ^4^Key Laboratory of Medical Electrophysiology, Ministry of Education & Medical Electrophysiological Key Laboratory of Sichuan, Institute of Cardiovascular Research, Southwest Medical University, Luzhou, Sichuan 646000, China; ^5^Department of Anesthesiology, Hospital (T.C.M) Affiliated to Southwest Medical University, Luzhou, Sichuan 646000, China

## Abstract

Age is the major risk factor for most of the deadliest diseases. Developing small molecule drugs with antiaging effects could improve the health of aged people and retard the onset and progress of aging-associated disorders. Bioactive secondary metabolites from medicinal plants are the main source for development of medication. Orientin is a water-soluble flavonoid monomer compound widely found in many medicinal plants. Orientin inhibits fat production, antioxidation, and anti-inflammatory activities. In this study, we explored whether orientin could affect the aging of *C. elegans*. We found that orientin improved heat, oxidative, and pathogenic stress resistances through activating stress responses, including HSF-1-mediated heat shock response, SKN-1-mediated xenobiotic and oxidation response, mitochondria unfolded responses, endoplasmic unfolded protein response, and increased autophagy activity. Orientin also could activate key regulators of the nutrient sensing pathway, including AMPK and insulin downstream transcription factor FOXO/DAF-16 to further improve the cellular health status. The above effects of orientin reduced the accumulation of toxic proteins (*α*-synuclein, *β*-amyloid, and poly-Q) and delayed the onset of neurodegenerative disorders in AD, PD, and HD models of *C. elegans* and finally increased the longevity and health span of *C. elegans*. Our results suggest that orientin has promising antiaging effects and could be a potential natural source for developing novel therapeutic drugs for aging and its related diseases.

## 1. Introduction

The longevity of human has improved dramatically during the past century due to improved healthcare and nutrition. However, age is the major cause of most life-threatening diseases, including cancer, cardiovascular diseases, neurodegenerative disorder, diabetes, and osteoporosis [1, 2]. The population older than 65 years is increasing fast in over the world [3]. So the rise of the population struggling with aging-associated disorders becomes an emerging socioeconomic challenge. Delaying the rate of biological aging would postpone the onset and progression of most age-related disorders. Therefore, the intervention of aging would be more effective than the treatment of the particular chronic disorders [4]. One of the major strategies is to develop small molecule drugs with antiaging effects. The current preclinical and clinical results have shown that some drugs have promising antiaging effects, such as rapamycin, senolytics, and metformin [4].

**Figure 1:**
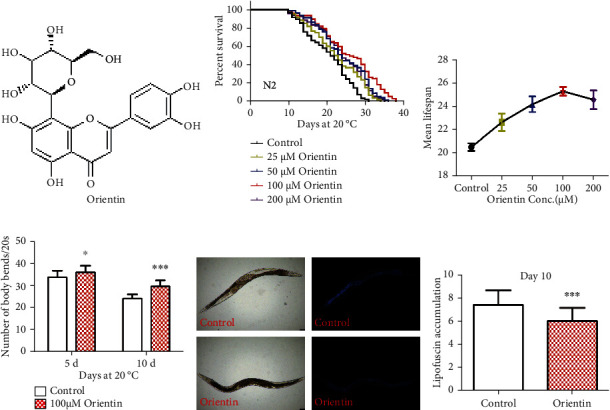
Orientin increases the longevity of *C. elegans* and slows the aging-related phenotypes. (a) The chemical structure of orientin. (b) The survival curves of the wild-type (N2) worms cultured at 20°C on NGM plates containing 0, 25, 50, 100, and 200 *μ*M of orientin, respectively. (c) Dose-response analysis of the effect of orientin on the longevity in *C. elegans*. The assays were independently performed at least three times. (d) Aging-related movements of N2 worms treated with or without 100 *μ*M of orientin. The mean body bending is in Table S2. (e) The intestinal autofluorescence of lipofuscin was analyzed on the 10th day of adulthood. The results of the mean lipofuscin accumulated are summarized in Table S3. Life span was analyzed by using the SPSS package and Kaplan-Meier, and *p* values were calculated by using the log-rank test. These results are represented as mean ± standard error of the mean (SEM), *p* < 0.05 was considered statistically significant, and detailed life span values are presented in Table S1 (supplementary information).

Bioactive secondary metabolites from medicinal plants are the main source for the development of medication. Orientin is one of the flavonoid compounds, widely existing in many plants, including nasturtium, bamboo leaves, and black fern (Figure 1(a)) [5]. Modern pharmacology research revealed that orientin has antioxidant activity, could reduce the H_2_O_2_-induced damages, enhance the detoxification capability, and improve the health status of D-galactose-aged mice [6, 7]. Orientin may suppress inflammatory responses [8, 9]. Orientin has antiadipogenesis activity through suppressing C/ebp expression and the phosphoinositide 3-kinase/Akt-FOXO1 signaling in adipocytes [10, 11]. Orientin could suppress the proliferation of MCF-7 breast cancer cells and colonic cells [12, 13]. Orientin was reported to have neuroprotective and cardiovascular protective effects [14–16].

Aging research has been extensively conducted in *Caenorhabditis elegans* (*C. elegans*) for their transparent body and short life span (about three weeks). Genetically identical individuals could be easily collected. An available whole-genome RNAi library and a large amount of genetic mutants facilitate the mechanistic analysis. Given the multiple pharmacological activities, especially the antioxidant activity of orientin, we are wondering whether orientin has geroprotective activities. In this study, we investigated the effect and mechanism of orientin on the longevity and neurodegenerative disorders in models of *C. elegans*.

## 2. Materials and Methods

### 2.1. Chemicals and Strains of *C. elegans*

Worms were provided by the Caenorhabditis Genetics Center (CGC) and maintained according to reports in literature [17]. The strains used in this study were as follows: N2 (Bristol, wild type), SJ4005 *zcIs4 [hsp-4::GFP] V*, SJ4100 *zcIs13V (hsp-6::GFP)*, LD1 *ldIs7 [skn-1B/C::GFP+pRF4 (rol-6(su1006))]*, CF1553 *muIs84 [(pAD76) sod-3p::GFP + rol-6(su1006)]*, NL5901 *([unc-54p::α-synuclein::YFP+unc-119(+)])*, BZ555 *egIs1(dat-1p::GFP)*, AM141 *(rmIs133)[unc-54p::Q40::YFP])*, CL4176 *dvIs27 [myo-3p::A-Beta (1-42)::let-851 3*′*UTR] + rol-6(su1006)] X*, JIN1375 *hlh-30(tm1978) IV*, DA1116 *eat-2(ad1116) II*, CB4876 *clk-1(e2519) III*, MQ887 *isp-1(qm150) IV*, TK22 *mev-1 (kn1) III*, RB754 *aak-2(ok524) X*, VC1027 *daf-15(ok1412)/nT1 IV; +/nT1* V, PS3551 *hsf-1(sy441) I*, CB1370 *daf-2(e1370) III*, TJ1052 *age-1(hx546) II*, RB759 *akt-1(ok525) V*, VC204 *akt-2(ok393) X*, CF1038 *daf-16(mu86) I*, EU1 *skn-1(zu67) IV*, VC199 *sir-2.1(ok434) IV*, VC893 *atg-18(gk378) V*, CF1903 *glp-1(e2141) III*, AA89 *daf-12 (rh274) X*, TJ356 *zIs356 [daf-16p::daf-16a/b::GFP + rol-6(su1006)] IV*, and BC12921 *[rCesT12G3.1::GFP + pCeh361].*

Worms were passaged at least for 2-3 generations preceding the formal assays. All worms were maintained at 20°C on NGM agar plates with *Escherichia coli* OP50 unless stated otherwise. The CL4176 strain was cultured at 15°C and switched to 25°C at the L3 stage in experiments [18]. The L1 larvae of the strain CF1903 were cultured at 20°C until grown into L4 larvae or young adults; then, worms were shifted to 25°C to inhibit the growth of germline and next transferred back to 20°C for experiments [19, 20].

The stock solution of chemicals including orientin, N-acetyl-cysteine (NAC), levodopa, and paraquat were made with deionized water. Before use, the solution of these compounds was sprayed on NGM plates and aired-dried overnight.

### 2.2. Longevity Assays

Longevity assays were conducted at 20°C unless otherwise stated. The synchronized late L4 larvae or young adults were transferred to plates seeded with inactive OP50 (65°C for 30 minutes), 20 *μ*M of 5-fluro-2′-deoxyuridine (FUDR, Sigma) to prevent the hatching of eggs, and the indicated compounds [21]. This day was defined as test day 0. Nematodes were then transferred to fresh corresponding plates with or without orientin every other day. The death of worms was monitored each day throughout the experiments. If the nematode cannot respond to this external mechanical stimulus (lightly touch the head or tail of the nematode with platinum), it is defined as dead. Worms under situations, such as having escaped from the plate, raised genital pores of nematodes, overflow of intestinal contents, or hatching of larvae from the body, are not counted as dead [21]. The final counted number of nematodes in each group is guaranteed to be at least 60. The life span experiments were conducted independently at least for three times. Statistical analyses were carried out by SPSS and Kaplan-Meier, and the statistical significance (*p* value) was calculated by the log-rank (Mantel-Cox) test.

### 2.3. Aging-Related Phenotype Analysis

For the body bending assay [22], worms were maintained as described in the longevity assay. Before counting, worms were transferred to M9 buffer and let stand for 15 seconds at 20°C; then, we scored the bending activity of the body for 20 seconds on the 5th and 10th day of adulthood under a stereomicroscope.

For the lipofuscin accumulation assay [23], synchronized worms were maintained for 7 days as described in the life span assay. The intestinal autofluorescence of lipofuscin was captured with a fluorescence microscope (Leica DFC 7000T) and analyzed by using ImageJ software.

Each of the above assays includes at least three independently replicated experiments. The significance (*p* values) was determined by the *t*-test.

### 2.4. Stress Resistance Assays

The total number of animals of the stress resistance analysis was at least 60 in each group, and these experiments were performed independently at least three times. Synchronized N2 late 4 larvae or young adults (*n* ≥ 100) were pretreated with 100 *μ*M of orientin for 7 days at 20°C on NGM plates before stress resistance assays. In the heat shock assay [24], the temperature at day 7 was upshifted to 35°C, and the dead individuals were identified by the touch with the platinum wire pick every 2 hours.

In the oxidative stress assay [21], the adult worms at day 7 were transferred to NGM plates containing 20 mM of paraquat (Sigma). The death of individuals was monitored every day.

In the pathogen resistance assay [25], the worms at late 4 larvae or young adults (*n* ≥ 100) were transferred to the plates seeded with live bacteria *Pseudomonas aeruginosa* (PA14) and cultured overnight before use. The death of individuals was monitored every day.

### 2.5. Neurodegenerative Disease Assay

The strain NL5901 expressing human *α*-synuclein fused with yellow fluorescent protein (YFP) in muscle cells was used as the model of Parkinson's disease (PD). First, worms were treated with 100 *μ*M of orientin for 7 days at 20°C; then, the fluorescent intensity was photographed by using a fluorescence microscope (Leica DFC 7000T) and analyzed by using the software ImageJ. The body bending assay was also analyzed as previously described [26].

The transgenic strain BZ555 expressing GFP (green fluorescent protein) in head neurons was also used as the model of PD [27, 28]. Synchronized late L3 larvae were incubated in the solution containing bacteria OP50, 50 *μ*M of 6-OHDA, and 10 mM of ascorbic acid for 1 hour at 20°C. Next, these nematodes were collected and incubated with or without drugs (the experimental group is 100 *μ*M of orientin, and the positive control group is 2 mM of levodopa) for 72 hours at 20°C. Lastly, the pictures of head neurons were captured by using a fluorescence microscope (Leica DFC 7000T) under the GFP channel. The intensity of GFP was calculated by using ImageJ. The individuals included in each assay were no less than 30. The assays were performed independently at least three times. *p* values were determined by the *t*-test.

The transgenic strain CL4176 expressing human amyloid-*β* protein was cultured on NGM at 15°C [18]. Under experiments [29], L3 larvae were incubated at 25°C to induce the expression of A*β*. The nematodes were considered to be paralyzed if they could not move their body when touched with a worm pick. Paralyzed nematodes were scored every 2 hours. The number of worms was at least 60 in each group. The assays were performed not less than three times. Statistical analyses were carried out according to longevity assays.

For the poly-Q aggregation assay in the model of Huntington's disease (HD), late 4 larvae or young adults of the strain AM141 were cultured with orientin for 7 days at 20°C. Pictures were taken by using a fluorescence microscope (Leica DFC 7000T) and examined by using ImageJ. The number of worms was at least 30 in each group. The assays were conducted independently at least three times. The statistical significance (*p* value) was determined by the *t*-test.

### 2.6. Oil Red O Staining and Nile Red Staining Assay

The accumulation and distribution of lipid content in nematodes could be observed by Oil Red O staining. Nile red staining is mainly used to observe the size and distribution of lipid droplets in nematodes [30]. We cultured the worms at L1 larvae with orientin for 3 days at 20°C. Then, nematodes were collected and stained according to the Oil Red O staining kit or the Nile red staining kit. Then, the fluorescent photos were taken by using a fluorescence microscope (Leica DFC 7000T) and examined by using the software ImageJ. These pictures were at least 30 per group. The experiments contained at least three independently repeated experiments. The statistical significance (*p* value) was determined by the *t*-test.

### 2.7. DAF-16::GFP Translocation Assay

The transgenic strain TJ356 expresses DAF-16 conjugated with GFP. Late 4 larvae or young adult worms (*n* ≥ 30) were incubated with drugs for 48 hours at 20°C [21, 31]. The location of fluorescence was monitored by using a fluorescent microscope (DFC 7000T) every hour. The individuals included in each group were no less than 30. The assays were conducted for three times.

### 2.8. Reactive Oxygen Species (ROS) Assay

Late 4 larvae or young adults (*n* ≥ 100) were incubated with either orientin or N-acetylcysteine (NAC, 1 mM) for 7 days at 20°C and then subjected to 2 mM of the oxidant paraquat (PQ) [32]. After that, the worms were collected and incubated with 50 *μ*M of H_2_DCF-DA (2′7′-dichlorofluorescein diacetate) for 1 hour in the dark at 20°C [33]. At least 30 animals in each group were placed on agar plates, captured by using a Leica epifluorescence microscope (DFC 7000T), and imaged by using ImageJ. The experiments were performed independently at least three times. The *p* values were calculated by the *t*-test.

### 2.9. Protein Expression Quantification Assay

These strains SJ4005, SJ4100, LD1, CF1553, and BC12921 express green fluorescent conjugated proteins HSP-4::GFP, HSP-6::GFP, SKN-1::GFP, SOD-3::GFP, and SQST-1::GFP, respectively. Late L4 larvae or young adults were transferred to the plates with or without orientin and maintained at 20°C for 7 days, except the strain BC12921, which was cultured for 3 days. Then, the individuals were collected and photographed by using a fluorescence microscope (DFC 7000T) and were measured by using the software ImageJ [34]. At least 30 individuals in each group were analyzed. Each experiment was performed independently for at least three times. The statistical significance (*p* values) was determined by the *t*-test.

### 2.10. ADP : ATP Ratio Quantification

The ADP : ATP ratio assay was performed as described previously [35]. In brief, L1 larvae were treated with orientin at 20°C for 3 days. Then, worms were collected and suspended with 2 mM of boiling MgSO_4_. After that, the worms were centrifuged and dried and then smashed for 10 minutes with a cell disruptor. The supernatant was collected, filtered, and analyzed by reverse-phase HPLC. Samples were separated in a Zorbax SB-C18 (250∗4.6 mm, 5 *μ*m) column by a flowing solution containing 5% of buffer A (100% MeOH) and 95% of buffer B (0.043 mol/L ammonium acetate). Nucleotide was detected at 254 nm [35].

### 2.11. Quantitative RT-PCR Assay

About 4000 young adult worms were treated with orientin at 20°C for 24 hours. Then, the worms were collected for RNA extraction by using the RNAiso Plus kit (Takara). The RNA was subsequently transformed into cDNA by using the High-Capacity cDNA Reverse Transcription Kit (Applied Biosystems). After that, the cDNA and corresponding primers were added into the Power SYBR Green PCR Master Mix (Applied Biosystems) and incubated by using the QuantStudio 6 Flex system. The relative mRNA levels of genes were carried out using the 2^–ΔΔCT^ method and normalized to the mRNA levels of the gene *cdc-42* [21]. Partial quantitative RT-PCR primers used in this publication are listed in Table S10.

### 2.12. Statistical Analyses

Longevity analyses were carried out by using the SPSS package and Kaplan-Meier, and the statistical significance (*p* values) was determined by using the log-rank test. Other results are expressed as the mean ± SD. The *p* values were determined by the two-tailed *t*-test. The results subjected to comparison was considered to have significant difference when *p* < 0.05.

## 3. Results

### 3.1. Orientin Increases the Longevity of *C. elegans*

To investigate whether orientin regulates the longevity of *C. elegans*, the wild-type N2 worms were treated with orientin with concentration ranging from 0 to 200 *μ*M. We found that orientin increased the longevity of *C. elegans* in a dose-dependent manner and 100 *μ*M of orientin increased the longest longevity of N2 worms by up to 22.2% (*p* < 0.001) (Figures 1(b) and 1(c), Table S1).

### 3.2. Orientin Slows the Aging-Related Phenotypes

Studies have shown that the athletic ability of worms reduces with aging [21]. To investigate whether orientin has the effect of delaying the decline of the body bending movement of worms with aging, we analyzed the body behavior of nematodes. The results showed that although the body swing frequency decreases with aging, orientin significantly reduced the decline of the body bending with aging (*p* < 0.001) (Figure 1(d), Table S2). Intestine autofluorescence indicates lipofuscin accumulation, which is one of the aging-related phenotypes [36, 37]. We found that orientin treatment reduced the fluorescence intensity of intestinal lipofuscin in nematodes by 18.7% compared with the control group (*p* < 0.001) (Figure 1(e), Table S3).

### 3.3. Orientin Improves the Stress Resistance of *C. elegans*

The long-lived nematodes usually show higher resistance to adverse stimuli from the external environment [38]. To test whether orientin could increase the stress resistance of worms, the N2 worms were pretreated with 100 *μ*M of orientin for 7 days at 20°C, followed by heat stress (35°C) or oxidative stress (20 mM of paraquat). For the pathogen stress assay, late L4 larvae or young adults were incubated on NGM plates seeded with live PA14 in the absence (0 *μ*M) or presence (100 *μ*M) of orientin.

**Figure 2:**
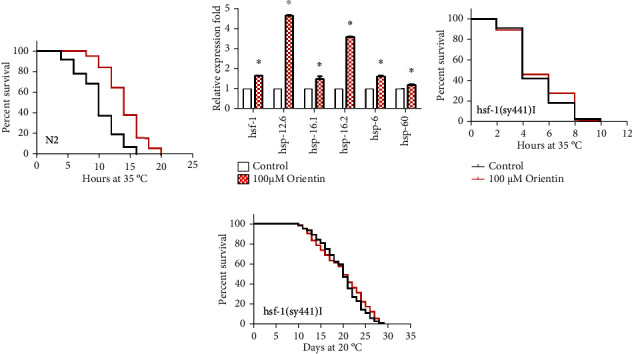
Orientin improves the resistance of heat stress. (a) The survival curves of N2 worms at 35°C. The detailed results are shown in Table S4. (b) QPCR analyses of the targeted genes of *hsf-1* (*hsp-12.6*, *hsp-16.1*, *hsp16.2*, *hsp-6*, and *hsp-60*) and itself in the wild-type N2 worms exposed to 100 *μ*M of orientin. The detailed results are in Table S9. (c) The survival curves of PS3551 *hsf-1(sy441) I* at 35°C. The detailed results are shown in Table S4. (d) The survival curves of PS3551 treated with or without 100 *μ*M of orientin at 20°C. The statistical details of the mutant with error bars representing SEM are presented in Table S4.

In the heat shock assay, we found that orientin could enhance the survival rate of *C. elegans* by 43.5% at 35°C of heat stress (*p* < 0.001) (Figure 2(a), Table S4). The heat shock factor protein HSF-1 regulates heat shock response and aging in *C. elegans* [39]. Orientin increased the transcription levels of the gene *hsf-1* and its regulated genes encoding heat shock proteins (HSPs), including *hsp-12.6*, *hsp-16.1*, *hsp-16.2*, *hsp-6*, and *hsp-60* (*p* < 0.05) (Figure 2(b), Table S9) [40]. To detect whether orientin could extend the longevity of worms via HSF-1, we analyzed the longevity of the *hsf-1* mutant PS3551 *hsf-1(sy441) I* cultured on the NGM plates treated with 100 *μ*M of orientin at 35°C or 20°C and found that orientin could not significantly prolong the longevity of *hsf-1* mutant in both conditions (*p* > 0.05) (Figures 2(c) and 2(d), Table S4).

**Figure 3:**
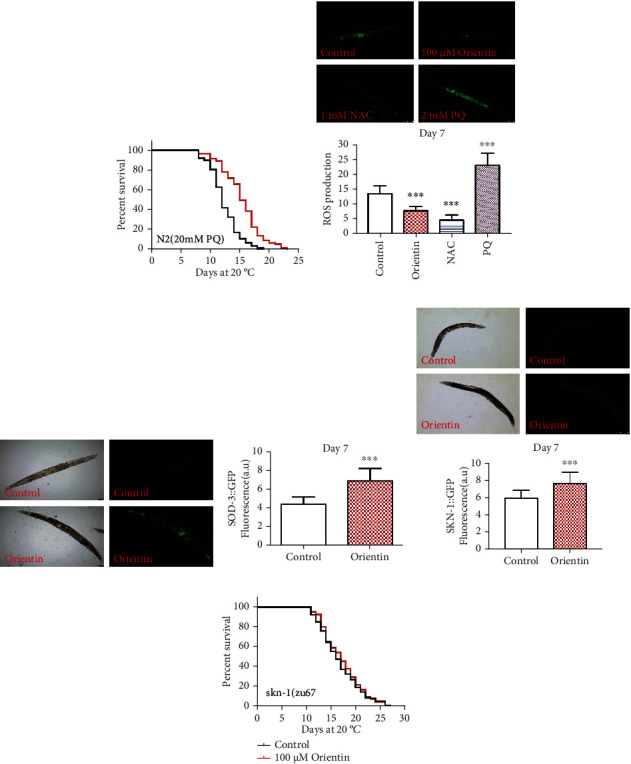
Orientin enhances the antioxidant capacity of *C. elegans*. (a) The survival curves of N2 nematodes exposed to paraquat (20 mM) and orientin (100 *μ*M); then, the death of individuals was counted every day and is summarized in Table S4. (b) Quantitation of intracellular level of ROS in N2 worms. The positive control is 2 mM of PQ (paraquat), and the negative control is 1 mM of NAC (N-acetyl-cysteine). (c) The accumulation of SOD-3::GFP in CF1553 treated with or without orientin for 7 days. (d) The representative pictures of SKN-1::GFP in LD1 transgenic worms treated with or without 100 *μ*M of orientin for 7 days. These pictures were photographed by using a fluorescence microscope (Leica DFC 7000T) and examined by using the software ImageJ. (e) The survival curves of EU1 *skn-1(zu67) IV* treated with or without medicine (100 *μ*M of orientin) at 20°C. Statistical details and repeats of these assays are presented in Tables S4 and S5.

Reactive oxygen species (ROS) generated in mitochondria is one of the major causes for many diseases and aging [41]. In the oxidative stress assay, our results showed that orientin enhanced the survival of worms exposed to oxidant paraquat by 23.3% (*p* < 0.001) (Figure 3(a), Table S4). Orientin significantly reduced the level of ROS in the body (*p* < 0.001) (Figure 3(b), Table S5). SOD-3 is a mitochondrial superoxide dismutase for antioxidation [42]. We examined the GFP intensity of SOD-3::GFP in the transgenic strain CF1553 and found that orientin significantly improved the GFP intensity (*p* < 0.001) (Figure 3(c), Table S5). The transcription factor SKN-1 is the main regulator for oxidative stress response. To investigate whether orientin activate SKN-1 to protect worms from oxidative damage, the accumulation of SKN-1::GFP was examined. We found that orientin treatment significantly enhanced the expression of SKN-1 (*p* < 0.001) (Figure 3(d), Table S5). Thus, we further determined whether orientin requires SKN-1 to prolong the longevity of *C. elegans*. We found that orientin could not increase the longevity of the loss-of-function mutant EU1 *skn-1(zu67) IV* (Figure 3(e), Table S7).

**Figure 4:**
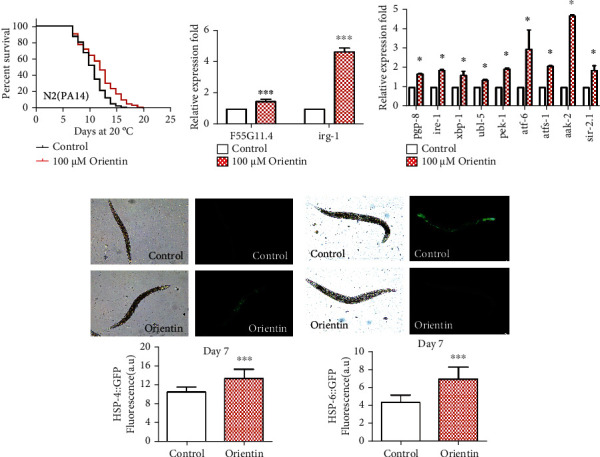
Orientin improves the resistance of pathogenic stress and increases the expression of heat shock proteins. (a) The survival curves of N2 worms fed with *P. aeruginosa* (PA14). (b) The mRNA expression levels of immune-related genes *F55G11.4* and *irg-1*. (c) The mRNA expression levels of genes *pgp-8*, *ire-1*, *xbp-1*, *ubl-5*, *pek-1*, *atf-6*, *atfs-1*, *aak-2*, and *sir-2.1* in the nutrition sensing signal pathway. (d) The pictures of green fluorescence in the transgenic strain SJ4005 expressing HSP-4 were captured by using a fluorescence microscope (Leica DFC 7000T) and analyzed by using the software ImageJ. (e) The image and quantitation of the protein HSP-6 in SJ4100 worms. The detailed results are presented in Tables S4, S5, and S9. Each of these assays was repeated independently at least three times (mean ± SD; Student's *t*-test; *n* ≥ 20; ^∗^*p* < 0.05, ^∗∗^*p* < 0.01, and ^∗∗∗^*p* < 0.001).

In the pathogen resistance assay, late L4 larvae or young adults were fed with pathogenic bacteria PA14. We found that orientin could enhance the survival of worms by up to 13.0% (*p* < 0.001) (Figure 4(a), Table S4). Orientin also significantly upregulated the mRNA levels of immune response genes *F55G11.4* and *irg-1* (*p* < 0.05) (Figure 4(b), Table S9) [43].

HSF-1, PEK-1, ATF6, XBP-1, IRE-1, ATFS-1 (a transcription factor of stress), and UBL-5 are the main regulators of cell proteostasis through mitochondrial and endoplasmic unfolded protein response. SIR-2.1 is an enzyme closely related to energy metabolism and regulates the life span and stress resistance of nematodes by binding to 14-3-3 protein and later activating DAF-16 [44]. In *C. elegans*, *aak-2* is an essential gene in the AMPK pathway and plays an important role in mitochondrial longevity regulation. We found that orientin could enhance the mRNA levels of genes in the above pathways, such as *pgp-8*, *ire-1*, *xbp-1*, *ubl-5*, *pek-1*, *atf-6*, *atfs-1*, *aak-2*, and *sir-2.1* (*p* < 0.05) (Figure 4(c), Table S9). Orientin also significantly increased the fluorescent intensity in worms expressing molecular chaperone heat shock proteins HSP-4::GFP and HSP-6::GFP (*p* < 0.001) (Figures 4(d) and 4(e), Table S5).

**Figure 5:**
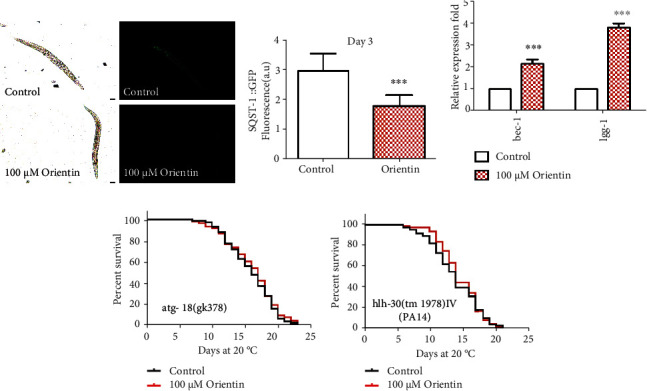
Orientin enhances autophagy activity. (a) The fluorescence intensity of SQST-1::GFP was analyzed at day 3 adulthood. The results with error bars representing SD are presented in Table S5. (b) The mRNA expression levels of genes *bec-*1 and *lgg-1* expressed in autophagy. The columns represent the mean value of three independent experiments with error bars representing SD in Table S9. (c) The life span analysis of *atg-18* mutant treated with or without 100 *μ*M of orientin. The statistical details of the mutant with error bars representing SEM are presented in Table S7. (d) The survival curves of JIN1375 *hlh-30(tm1978) IV* treated with or without orientin (100 *μ*M) at 20°C. The statistical details of the mutant with error bars representing SEM are summarized in Table S4. Each of these experiments was repeated independently at least three times.

Autophagy is responsible for xenobiotic and misfolded protein degradation. The decrease in the autophagy substrate P62/SQST-1 indicates the increased activity of autophagy [45, 46]. We observed that orientin decreased the fluorescence intensity of SQST-1::GFP by 35.39% (*p* < 0.001) (Figure 5(a), Table S5). We also observed the increased mRNA levels of autophagy expressing genes *bec-1* and *lgg-1* [45] (*p* < 0.05) (Figure 5(b), Table S9). The gene *atg-18* is necessary for the recovery and recycling of vesicles in autophagy [47]. The gene *hlh-30* regulates autophagy activity, lysosomal biogenesis, and innate immune response [43]. Our results showed that orientin could not prolong the longevity of the loss-of-function mutants VC893 *atg-18(gk378) V* (Figure 5(c), Table S7) and JIN1375 *hlh-30(tm1978) IV* (Figure 5(d), Table S4). In summary, orientin could improve the resistance of nematodes to heat, oxidative, and pathogenic bacterial stress through antioxidative and unfolded protein response to maintain proteostasis.

### 3.4. Orientin Could Postpone the Development of Neurodegenerative Disorders in Models of *C. elegans*

The accumulation of misfolded proteins could be toxic and lead to malfunction and eventually death of neurons. The development of neural pathology with aging causes a variety of neurodegenerative disorders, including PD, AD, and HD [48, 49]. We used *C. elegans* models to study whether orientin has a protective effect on these diseases.

**Figure 6:**
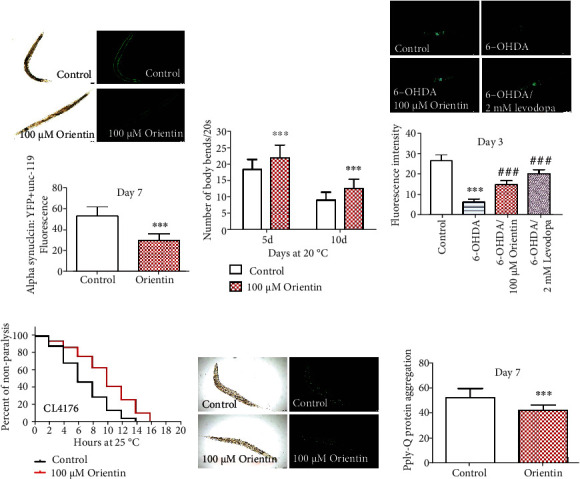
Orientin delays the progression of neurodegenerative diseases in models of *C. elegans*. (a) The aggregation of *α*-synuclein in NL5901 treated with 100 *μ*M of orientin was captured with a fluorescence microscope (Leica DFC 7000 T) and analyzed by using the software ImageJ. The results are presented in Table S5. (b) Aging-related movements of NL5901 on the 5th and 10th days. The mean body bending could be found in Table S6. (c) T5he fluorescence intensity of the head dopamine neurons in worms BZ555 rescued by orientin and NAE after 6-OHDA induction. After the induction of 6-OHDA, the fluorescence intensity was significantly reduced. 30 worms per condition were analyzed in each independent test, and the statistical details and the results of three repeated experiments are presented in Table S5. (d) The paralysis counts of the transgenic strain CL4176 at 25°C. Error bars represent SEM. The mean life span of CL4176 is summarized in Table S7. (e) The poly-Q protein aggregation of AM141 with or without orientin (100 *μ*M); the detailed results are summarized in Table S5. These experiments were each performed at least three times (mean ± SD; Student's *t-*test; *n* ≥ 30; ^∗^*p* < 0.05, ^∗∗^*p* < 0.01, and ^∗∗∗^*p* < 0.001).

PD is a common fatal neurological disease that gradually worsens with aging due to the progressive aggregation of *α*-synuclein in neurons, especially the damage of dopaminergic (DA) neurons [26, 27]. So, we tested the accumulation of *α*-synuclein and the body bending behavior in the transgenic NL5901worms. We found that the orientin reduced the accumulation of *α*-synuclein by 43.6% and slowed the decline of the body bending with aging (*p* < 0.001) (Figures 6(a) and 6(b), Tables S5 and S6). The DA neurons of worm BZ555 could be degenerated by a liquid medium containing 50 mM of 6-OHDA. The degeneration of neurons could be determined by using a fluorescent photograph. We found that the mean fluorescence intensity of neurons in the strain BZ555 was decreased from 26.793 ± 2.846 to 6.301 ± 1.520 after exposure to 6-OHDA. Orientin and the positive drug levodopa treatment increased the mean fluorescence intensity to 14.953 ± 1.952 and 20.398 ± 1.874 from exposing to 6-OHDA, respectively (Figure 6(c), Table S5).

The typical pathological changes of AD include the brain plaques formed by the accumulation of amyloid (A*β*) and neurofibrillary tangles (NFTs) formed by aggregation of abnormally phosphorylated tau [50]. CL4176 worms express human A*β*1-42 in the cytoplasm of body wall muscle cells at 25°C and become paralyzed [48]. Our results showed that orientin could postpone the onset of paralysis in the strain CL4176 (*p* < 0.001) (Figure 6(d), Table S7).

As the model of Huntington's disease (HD), the AM141 (*rmIs133) [unc-54p::Q40::YFP]* worms express polyglutamine fused with YFP [51]. We found that orientin reduced the aggregation of poly-Q by 19.1% at day 7 (*p* < 0.001) (Figure 6(e), Table S5). In summary, we found that orientin has an inhibitory effect on age-related neurodegenerative diseases (PD, AD, and HD).

### 3.5. Orientin Depends on FOXO/DAF-16 to Prolong the Longevity of *C. elegans*

**Figure 7:**
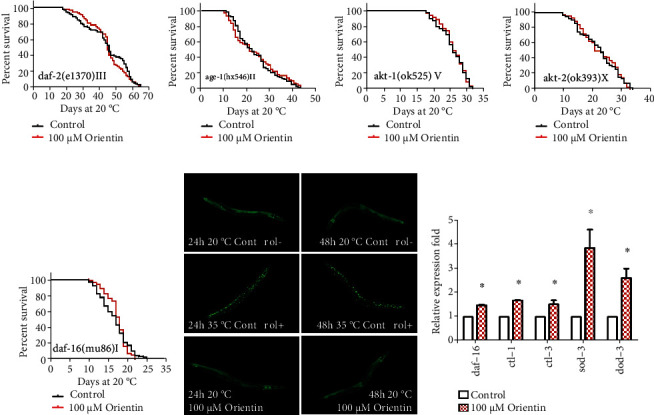
Orientin depends on FOXO/DAF-16 to prolong the longevity of *C. elegans*. (a–e) The survival curves of *daf-2*, *age-1*, *akt-1*, *akt-2*, and *daf-16*, mutants in the absence or presence of orientin (100 *μ*M). These results are represented as mean ± standard error of the mean (SEM). The results were considered statistically significant when *p* < 0.05. Statistical details of the longevity of the mutants and repeats of these experiments are presented in Table S7. (f) Effect of orientin on the nuclear localization of DAF-16. The representative fluorescence photomicrograph of transgenic TJ356 worms with cytosolic, intermediary, and nuclear staining. (g) The mRNA expression levels of genes downstream of *daf-16* (*ctl-1*, *ctl-3*, *sod-3*, and *dod-3*) and itself in N2 worms exposed to orientin (100 *μ*M) versus control worms. The columns are shown in Table S9.

The transcription factor FOXO/DAF-16 is the central regulator of life span, stress response, development, reproduction, and metabolism [49]. In *C. elegans*, insulin or insulin-like ligands bind to the DAF-2/insulin (tyrosine kinase) receptor, which then phosphorylates the phosphatidylinositol 3 kinase (encoded by *age-1*) and generates PIP3. PIP3 activates kinases such as SGK-1 and AKT-1/2 through phosphorylation of PDK-1. Finally, AKT-1/2 phosphorylates and prevents DAF-16 from entering the nucleus to initiate transcription of downstream genes [49, 52]. Our results showed that orientin could not prolong the longevity of loss-of-function mutant CF1038 *daf-16(mu86) I* and the long-lived mutants CB1370 *daf-2(e1370) III*, TJ1052 *age-1(hx546) II*, RB759 *akt-1(ok525) V*, and VC204 *akt-2(ok393) X* (*p* > 0.05) (Figures 7(a)–7(e), Table S7).

To identify whether orientin could promote nuclear localization of DAF-16, we observed the subcellular localization of DAF-16 conjugated with GFP in the strain TJ356 treated with orientin. We found that orientin could not increase the amount of DAF-16 in the nucleus (Figure 7(f)). In addition, orientin increased the mRNA expression levels of DAF-16-regulated genes, such as *ctl-1*, *ctl-3*, *sod-3*, and *dod-3* (*p* < 0.05) (Figure 7(g), Table S9).

### 3.6. Orientin Affects the Metabolism of *C. elegans*

**Figure 8:**
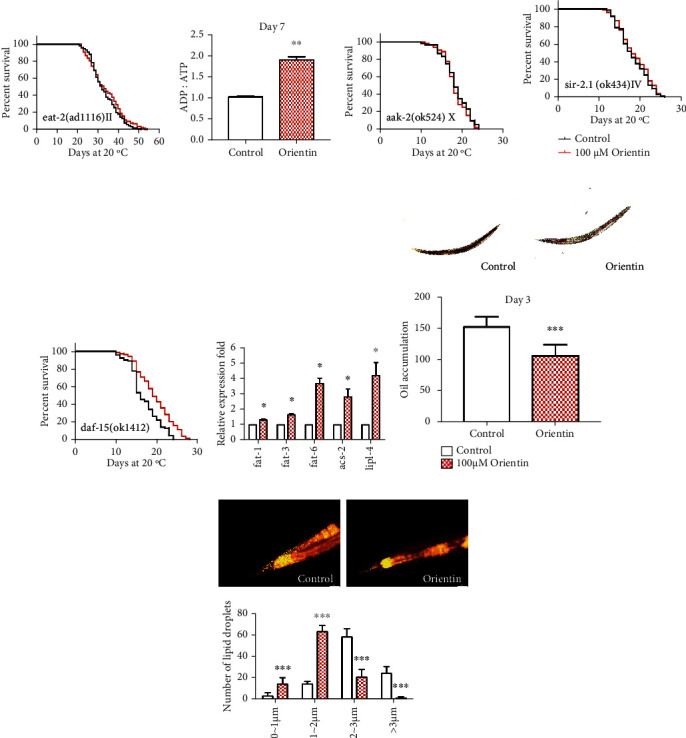
The effect of orientin on the energy metabolism and fat metabolism of *C. elegans*. (a) The survival curves of *eat-2* mutants in the NGM plates with or without orientin. The results are shown in Table S7. (b) The ADP : ATP ratio of N2 nematodes cultured with or without orientin at 20°C for 3 days was determined by HPLC. The figures are exhibited in Table S8. (c–e) The survival curves of *aak-2*, *sir-2.1*, and *daf-15* mutants untreated or treated with orientin (100 *μ*M) at 20°C. The statistical details of these mutants are presented in Table S7. (f) QPCR analysis of fat-related genes (*fat-1*, *fat-3*, *fat-6*, *acs-2*, and *lipl-4*) in the N2 worms treated with orientin (100 *μ*M) versus control worms. The columns are shown in Table S9. (g) The relative Oil Red O intensity of wild-type N2 worms treated or untreated with orientin for 3 days was calculated by using ImageJ. (h) The relative Nile red fluorescence intensity of N2 worms cultured with or without orientin for 3 days was analyzed by using ImageJ. These experiments were each performed at least three times (mean ± SD; Student's *t-*test; *n* ≥ 30; ^∗^*p* < 0.05, ^∗∗^*p* < 0.01, and ^∗∗∗^*p* < 0.001).

The pharyngeal pump dysfunction mutant DA1116 *eat-2(ad1116) II* is long-lived for reduced uptake of food. Our results showed that orientin could not further prolong the longevity of DA1116 (*p* = 0.166) (Figure 8(a), Table S7). AMPK is critical for the regulation of energy and life span. Reduced level of ATP activates the AMPK pathway [53]. We used HPLC to determine the ratio of ADP : ATP of nematodes treated with orientin. We found that orientin significantly increased the ratio of ADP : ATP (*p* = 0.0031) (Figure 8(b), Table S8). In *C. elegans*, *sir-2.1* is one of the genes encoding NAD^+^-dependent histone deacetylase, and its overexpression can extend the longevity [54]. We found that orientin could not increase the longevity of the mutant RB754 *aak-2(ok524) X* and VC199 *sir-2.1(ok434) IV* (*p* > 0.05) (Figures 8(c) and 8(d), Table S7). The target of rapamycin (TOR) protein is a kinase critical for nutrient regulation [55]. The main receptor of mTOR in *C. elegans* is encoded by *daf-15*. Our results showed that orientin could prolong the longevity of the mutant VC1027 *daf-15(ok1412)/nT1 IV; +/nT1 V)* by 14.8% (*p* < 0.05) (Figure 8(e), Table S7).

We detected fat content and lipid droplet size by the Oil Red staining and the Nile red staining. The results showed that orientin significantly reduced the fat content and lipid droplet size (*p* < 0.001) (Figures 8(g) and 8(h)). Moreover, we found that orientin increased the mRNA levels of lipid metabolism genes *fat-1*, *fat-3*, *fat-6*, *acs-2*, and *lipl-4* (*p* < 0.05) (Figure 8(f), Table S9).

**Figure 9:**
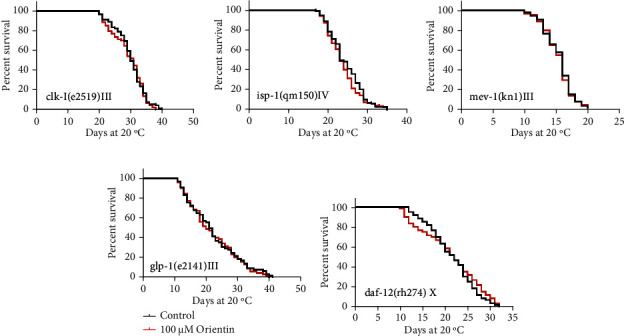
The effect of orientin on the mitochondrial and reproductive signaling pathway of *C. elegans*. (a–e) The survival curves of *clk-1*, *isp-1*, *mev-1*, *glp-1*, and *daf-12* mutants in the absence (0 *μ*M) or presence (100 *μ*M) of orientin. Orientin could not further extend the life span compared with the control group (*p* > 0.05). The statistical details of these mutants with error bars representing SEM are summarized in Table S7.

To explore whether orientin increases the longevity of *C. elegans* by acting on the mitochondrial signaling pathway, we studied the effect of orientin on the long-lived mitochondrial dysfunction mutants in genes *clk-1* (the homologous gene of human coenzyme Q7 hydroxylase) and *isp-1* (the Rieske iron-sulfur protein), as well as on the short-lived mutant in the gene *mev-1* (the cytochrome b large subunit (Cyt-1/ceSDHC)) [51]. Our results showed that orientin could not increase the longevity of CB4876 *clk-1(e2519) III*, MQ887 *isp-1(qm150) IV*, and TK22 *mev-1 (kn1) III* (*p* > 0.05) (Figures 9(a)–9(c), Table S7).

The reproductive system has strong impact on the metabolism and life span of *C. elegans* [56]. Gonadal stem cells promote fat storage and accelerate aging; gonadal glandular cells promote fat hydrolysis and delay aging [57]. To investigate whether orientin could affect the reproduction of nematodes, we selected the mutants CF1903 *glp-1(e2141) III* (whose gonad was destroyed at 25°C) and AA89 *daf-12 (rh274) X* (DAF-12 regulates the function of the reproductive system) to test if orientin acts on this pathway. Our results showed that orientin could not prolong the longevity of the two mutants (*p* > 0.05) (Figures 9(d) and 9(e), Table S7).

## 4. Discussion

Here, we investigated whether orientin could regulate the life span in *C. elegans*. We found that orientin significantly extended the longevity of *C. elegans*, postponed the slowing of body bending with aging, decrease the hoarding of lipofuscin and fat content, reduced the lipid droplet size in N2 worms, enhanced the stress resistance of nematodes, and delayed the progression of age-related diseases, including PD, AD, and HD. Our results support the previous report that orientin has antioxidative and antiaging activity in D-galactose-aged mice [7], suggesting that orientin has promising antiaging effects and is expected to become a potential source for developing novel therapeutic drugs for aging and its related diseases.

Various signals including the IIS insulin pathway, TOR pathway, germline pathway, and hormetic pathway converge on the key transcription factor FOXO/DAF-16 to modulate metabolism, stress, and aging [49]. There are many ways to modulate the activity of DAF-16, such as phosphorylation, acetylation, methylation, or acting as a coactivator. Here, we showed that orientin could not increase the longevity of *C. elegans* without DAF-16. Orientin could induce the mRNA levels of DAF-16-regulated genes, although it could not translocate DAF-16 from the cytoplasm to the nucleus. Orientin also could not prolong the longevity of long-lived mutants in the IIS insulin pathway upstream of *daf-16*, indicating that either orientin acts on the IIS insulin pathway or the impact of orientin on longevity enhancement is not big enough to make a significant difference from the long-lived mutants.

Orientin improves the capability of resistance in *C. elegans* to stresses, such as heat, oxidation, and pathogenic bacteria. Further mechanistic investigation revealed that orientin activates transcription factors SKN-1, the master regulator of xenobiotic metabolism and oxidative response, and HSF-1, the crucial regulator of protein unfolded response [39]. SKN-1 is the homologue of Nrf2 in mammals, our results support the findings that orientin might reduce the cognitive malfunction and oxidative stress in AD mice by activating Nrf2 [58], and HSF-1 regulated genes encoding molecular chaperone HSPs. HSPs are associated with the extension of life span and antiaging in many organisms [59]. We showed that orientin could not significantly prolong the longevity of mutant *hsf-1(sy441) I* with or without heat stress. The bacterial pathogen *P. aeruginosa* produces multiple toxins that perturb host protein synthesis and mitochondrial function, including perturbed proteostasis and OXPHOS impairment [60]. Antibacterial response involves mitochondrial unfolded response, endoplasmic stress response, autophagy activation, xenobiotic metabolism, and oxidative response. The transcription factor ATF-6 activates the expression of genes regulating UPR [61]. Upon unfolded protein stress, the endoplasmic reticulum (ER) transmembrane protein IRE-1 splices *xbp-1* mRNA to initiate the production of the transcription factor XBP-1 [62]. ATFS-1 (a transcription factor of stress) and UBL-5 are required for the mitochondrial unfolded protein response activation in *C. elegans* [63]. We showed that orientin could upregulate the mRNA levels of genes regulating the unfolded protein response (UPR), such as *pek-1* (encoding eukaryotic translation initiation factor 2-alpha kinase), *ire-1*, *xbp-1*, *ubl-5*, *atfs-1*, and *atf-6*. Orientin treatment also increased the GFP fluorescent intensity of the chaperone HSP-4 induced by *ire-1* and *xbp-1* through UPR^ER^ and the protein HSP-6 induced by *atfs-1* and *ubl-5* through the mitochondrial UPR. Furthermore, orientin could significantly increase the expression of the autophagy-related genes *bec-1* and *lgg-1* and decrease the content of SQST-1, the substrate of autophagy. These results suggest that orientin could activate multiple stress response pathways to maintain the cellular homeostasis, supporting the previous findings that orientin has the antiviral [64, 65], antibacterial [66, 67], and antiradiation activities [68, 69]. Our results also support the findings that orientin could mitigate colorectal lesions in rats by its antioxidative activity and induce detoxification enzymes regulated by Nrf2 [70].

Nutrient-processing pathways play a crucial role in aging. The gene *aak-2* is an essential gene in the AMPK pathway and an important regulator of longevity. Reducing the level of ATP could activate the AMPK pathway [53, 71]. We found that orientin treatment could significantly increase the ratio of ADP : ATP and the mRNA levels of genes in the nutrient sensing pathway, such as *pgp-8*, *atf-6*, *aak-2*, and *sir-2.1*. We show that orientin could not significantly prolong the longevity of mutants of genes in the nutrient-processing pathway, such as *eat-2*, *clk-1*, *isp-1*, *aak-2*, and *sir-2.1*. Collectively, the above results suggest that longevity extension of worms by orientin might involve the energy-processing pathways. It was reported that orientin could inhibit adipogenesis and intracellular triglyceride accumulation in 3T3-L1 cells by inhibiting the protein expressions of C/EBP*α* and PPAR*γ* [72, 73]. Here, we showed that orientin could significantly reduce the fat content and lipid droplet size in *C. elegans* probably by upregulating the expression of lipid metabolism genes. The mechanism of orientin on regulating lipid metabolism in mammals and *C. elegans* which might be conservative is worthy of further investigation.

In summary, we show that orientin could increase the longevity and healthy life span of *C. elegans* and increase stress resistances through nutrition sensing and cellular protective pathways (Figure S1). The activation of AMPK inhibits mTOR and its downstream proteins' translation process, contributing to reducing the production of toxic proteins (*α*-synuclein, *β*-amyloid, and poly-Q). Moreover, through activating mitochondrial, heat, oxidative, and pathogenic stress responses, including mitochondria unfolded responses, endoplasmic unfolded protein response, and increased autophagy activity, together with the increased capability to maintain proteostasis, the above effects of orientin delayed the development of neurodegenerative diseases, such as AD, PD, and HD, in models of *C. elegans*. Our findings support that orientin has multiple therapeutic effects, such as antioxidant, antiaging, antiviral, anti-inflammation, antiadipogenesis, cardioprotective, radioprotective, and neuroprotective [5]. Although orientin was water-soluble [74], pharmacokinetic studies have proven that orientin can be rapidly absorbed and transported to various organs and tissues in the body, with better absorption, faster clearance, and no accumulation of poisoning [75]. Therefore, in the future research, its pharmacological action mechanism should be studied in depth, including experiments in mammal animal models of aging and aging-related diseases for the beneficial effects of orientin in view of the translation into clinics.

## Data Availability

The article and supplementary materials contain all the data supporting the results of this research.

## Supplementary Materials

Supplementary Materials Figure S1: graphic abstract. Table S1: mean life span of wild-type *C. elegans* (N2) treated with orientin in different concentrations. Table S2: effect of orientin on body bending of wild-type *C. elegans* (N2). Table S3: effect of orientin on lipofuscin of wild-type *C. elegans* (N2). Table S4: effect of orientin on resistance to high temperature (35°C), oxidation (20 mM of paraquat), and bacteria (*Pseudomonas aeruginosa*) in N2 worms. Table S5: effect of orientin on protein expression shown as fluorescence intensity. Table S6: effect of orientin on body bending of NL5901 ([unc-54p::*α*-synuclein::YFP+unc-119(+)]). Table S7: mean life span of orientin on life span of *C. elegans* strains. Table S8: effect of orientin on the ratio of ADP : ATP in N2 worms. Table S9: effect of orientin on mRNA expression of wild-type *C. elegans* (N2). Table S10: primers used for the analysis of mRNA expression levels in N2 worms.

## References

[1] de Cabo R., Gutierrez D. C., Bernier M., Hall M., Madeo F. (2014). The search for antiaging interventions: from elixirs to fasting regimens. *Cell*.

[2] Kaeberlein M., Rabinovitch P. S., Martin G. M. (2015). Healthy aging: the ultimate preventative medicine. *Science*.

[3] Stegemann S., Ecker F., Maio M. (2010). Geriatric drug therapy: neglecting the inevitable majority. *Ageing Research Reviews*.

[4] Partridge L., Fuentealba M., Kennedy B. K. (2020). The quest to slow ageing through drug discovery. *Nature Reviews. Drug Discovery*.

[5] Lam K. Y., Ling A. P. K., Koh R. Y., Wong Y. P., Say Y. H. (2016). A review on medicinal properties of orientin. *Advances in Pharmacological Sciences*.

[6] Xiao Q. F., Piao R., Wang H. Y., Li C. H., Song L. S. (2018). Orientin-mediated Nrf2/HO-1 signal alleviates H_2_O_2_-induced oxidative damage via induction of JNK and PI3K/AKT activation. *International Journal of Biological Macromolecules*.

[7] An F., Yang G. D., Tian J. M., Wang S. H. (2012). Antioxidant effects of the orientin and vitexin in Trollius chinensis Bunge in D-galactose-aged mice. *Neural Regeneration Research*.

[8] Xiao Q. F., Qu Z. H., Zhao Y., Yang L. M., Gao P. J. (2017). Orientin ameliorates LPS-induced inflammatory responses through the inhibitory of the NF-*κ*B pathway and NLRP3 inflammasome. *Evidence-based Complementary and Alternative Medicine: Ecam*.

[9] Dhakal H., Lee S., Choi J. K., Kwon T. K., Khang D., Kim S. H. (2020). Inhibitory effects of orientin in mast cell-mediated allergic inflammation. *Pharmacological reports: PR*.

[10] Nagai S., Matsumoto C., Shibano M., Fujimori K. (2018). Suppression of fatty acid and triglyceride synthesis by the flavonoid orientin through decrease of C/EBP*δ* expression and inhibition of PI3K/Akt-FOXO1 signaling in adipocytes. *Nutrients*.

[11] Kwon J. H., Hwang S. Y., Han J. S. (2017). Bamboo (Phyllostachys bambusoides) leaf extracts inhibit adipogenesis by regulating adipogenic transcription factors and enzymes in 3T3-L1 adipocytes. *Food Science and Biotechnology*.

[12] Kim S. J., Pham T. H., Bak Y., Ryu H. W., Oh S. R., Yoon D. Y. (2018). Orientin inhibits invasion by suppressing MMP-9 and IL-8 expression via the PKC*α*/ERK/AP-1/STAT3-mediated signaling pathways in TPA-treated MCF-7 breast cancer cells. *Phytomedicine: international journal of phytotherapy and phytopharmacology*.

[13] Thangaraj K., Vaiyapuri M. (2017). Orientin, a c-glycosyl dietary flavone, suppresses colonic cell proliferation and mitigates NF-*κ*B mediated inflammatory response in 1,2-dimethylhydrazine induced colorectal carcinogenesis. *Biomedicine & pharmacotherapy = Biomedecine & pharmacotherapie*.

[14] Li F. F., Zong J., Zhang H. (2017). Orientin reduces myocardial infarction size via eNOS/NO signaling and thus mitigates adverse cardiac remodeling. *Frontiers in Pharmacology*.

[15] Jing S. Q., Wang S. S., Zhong R. M. (2020). Neuroprotection of Cyperus esculentus L. orientin against cerebral ischemia/reperfusion induced brain injury. *Neural Regeneration Research*.

[16] Guo D. D., Hu X. Y., Zhang H. J., Lu C. H., Cui G. W., Luo X. J. (2018). Orientin and neuropathic pain in rats with spinal nerve ligation. *International Immunopharmacology*.

[17] Brenner S. (1974). The genetics of *Caenorhabditis elegans*. *Genetics*.

[18] Xu J., Yuan Y., Zhang R. (2019). A deuterohemin peptide protects a transgenic *Caenorhabditis elegans* model of Alzheimer’s disease by inhibiting A*β*1–42 aggregation. *Bioorganic Chemistry*.

[19] Hsin H., Kenyon C. (1999). Signals from the reproductive system regulate the lifespan of *C. elegans*. *Nature*.

[20] Wan Q. L., Meng X., Fu X. D. (2019). Intermediate metabolites of the pyrimidine metabolism pathway extend the lifespan of C. elegans through regulating reproductive signals. *Aging*.

[21] Zheng S. Q., Huang X. B., Xing T. K., Ding A. J., Wu G. S., Luo H. R. (2017). Chlorogenic acid extends the lifespan of *Caenorhabditis elegans* via insulin/IGF-1 signaling pathway. *The journals of gerontology. Series A, Biological sciences and medical sciences*.

[22] Huang C., Xiong C. J., Kornfeld K. (2004). Measurements of age-related changes of physiological processes that predict lifespan of *Caenorhabditis elegans*. *Proceedings of the National Academy of Sciences of the United States of America*.

[23] Aranaz P., Peñ A., Vettorazzi A. (2021). Grifola frondosa (Maitake) extract reduces fat accumulation and improves health span in C. elegans through the DAF-16/FOXO and SKN-1/NRF2 signalling pathways. *Nutrients*.

[24] Wilson M. A., Hale B. S., Kalt W., Ingram D. K., Joseph J. A., Wolkow C. A. (2006). Blueberry polyphenols increase lifespan and thermotolerance in *Caenorhabditis elegans*. *Aging Cell*.

[25] Pees B., Kloock A., Nakad R., Barbosa C., Dierking K. (2017). Enhanced behavioral immune defenses in a *C. elegans* C-type lectin-like domain gene mutant. *Developmental and Comparative Immunology*.

[26] Maulik M., Swarup M., Ito A. B., Taylor B. E., Vayndorf E. M. (2017). Behavioral phenotyping and pathological indicators of Parkinson's disease in *C. elegans* models. *Frontiers in Genetics*.

[27] Che X., Barclay J. W., Burgoyne R. D., Morgan A. (2015). Using *C. elegans* to discover therapeutic compounds for ageing-associated neurodegenerative diseases. *Chemistry Central Journal*.

[28] Tsai C. W., Tsai R. T., Liu S. P. (2017). Neuroprotective effects of betulin in pharmacological and transgenic *Caenorhabditis elegans* models of Parkinson's disease. *Cell Transplantation*.

[29] Link C. D., Taft A., Kapulkin V. (2003). Gene expression analysis in a transgenic *Caenorhabditis elegans* Alzheimer 's disease model. *Neurobiology of Aging*.

[30] Fouad A. D., Pu S. H., Teng S. (2017). *Caenorhabditis elegans* quantitative assessment of fat levels in using dark field microscopy. *G3 (Bethesda, Md.)*.

[31] Rangsinth P., Prasansuklab A., Duangjan C. (2019). Leaf extract of Caesalpinia mimosoides enhances oxidative stress resistance and prolongs lifespan in *Caenorhabditis elegans*. *BMC Complementary and Alternative Medicine*.

[32] Xiong L. G., Chen Y. J., Tong J. W., Gong Y. S., Huang J. A., Liu Z. H. (2018). Epigallocatechin-3-gallate promotes healthy lifespan through mitohormesis during early-to-mid adulthood in *Caenorhabditis elegans*. *Redox Biology*.

[33] Duangjan C., Rangsinth P., Gu X. J., Wink M., Tencomnao T. (2019). Lifespan extending and oxidative stress resistance properties of a leaf extracts from L. in *Caenorhabditis elegans*. *Oxidative medicine and cellular longevity*.

[34] Huang X. B., Mu X. H., Wan Q. L., He X. M., Wu G. S., Luo H. R. (2017). Aspirin increases metabolism through germline signalling to extend the lifespan of *Caenorhabditis elegans*. *PLoS One*.

[35] Wan Q. L., Zheng S. Q., Wu G. S., Luo H. R. (2013). Aspirin extends the lifespan of *Caenorhabditis elegans* via AMPK and DAF-16/FOXO in dietary restriction pathway. *Experimental Gerontology*.

[36] Liao V. H. C., Yu C. W., Chu Y. J., Li W. H., Hsieh Y. C., Wang T. T. (2011). Curcumin-mediated lifespan extension in *Caenorhabditis elegans*. *Mechanisms of Ageing and Development*.

[37] Lin C. X., Zhang X. Y., Xiao J. (2019). Effects on longevity extension and mechanism of action of carnosic acid in *Caenorhabditis elegans*. *Food & Function*.

[38] Hamilton K. L., Miller B. F. (2016). What is the evidence for stress resistance and slowed aging?. *Experimental Gerontology*.

[39] Lu M., Tan L., Zhou X. G. (2020). Secoisolariciresinol diglucoside delays the progression of aging-related diseases and extends the lifespan of *Caenorhabditis elegans* via DAF-16 and HSF-1. *Oxidative Medicine and Cellular Longevity*.

[40] Labbadia J., Brielmann R. M., Neto M. F., Lin Y. F., Haynes C. M., Morimoto R. I. (2017). Mitochondrial stress restores the heat shock response and prevents proteostasis collapse during aging. *Cell Reports*.

[41] Lee H. C., Wei Y. H. (2001). Mitochondrial alterations, cellular response to oxidative stress and defective degradation of proteins in aging. *Biogerontology*.

[42] Shi Y. C., Yu C. W., Liao V. H. C., Pan T. M. (2012). Monascus-fermented dioscorea enhances oxidative stress resistance via DAF-16/FOXO in *Caenorhabditis elegans*. *PLoS One*.

[43] Kumar S., Egan B. M., Kocsisova Z. (2019). Lifespan extension in *C. elegans* caused by bacterial colonization of the intestine and subsequent activation of an innate immune response. *Developmental Cell*.

[44] Berdichevsky A., Viswanathan M., Horvitz H. R., Guarente L. (2006). *C. elegans* SIR-2.1 interacts with 14-3-3 proteins to activate DAF-16 and extend life span. *Cell*.

[45] Wei S., Chen W., Qin J. F. (2016). Multitarget-directed oxoisoaporphine derivatives: anti-acetylcholinesterase, anti-*β*-amyloid aggregation and enhanced autophagy activity against Alzheimer's disease. *Bioorganic & Medicinal Chemistry*.

[46] Yang Z. Z., Yu Y. T., Lin H. R., Liao D. C., Cui X. H., Wang H. B. (2018). *Lonicera japonica* extends lifespan and healthspan in *Caenorhabditis elegans*. *Free Radical Biology & Medicine*.

[47] Takacs Z., Sporbeck K., Stoeckle J., Carvajal M. J. P., Grimmel M., Proikas-Cezanne T. (2019). ATG-18 and EPG-6 are both required for autophagy but differentially contribute to lifespan control in *Caenorhabditis elegans*. *Cell*.

[48] Lublin A. L., Link C. D. (2013). Alzheimer's disease drug discovery: in vivo screening using *Caenorhabditis elegans* as a model for *β*-amyloid peptide-induced toxicity. *Drug discovery today: Technologies*.

[49] Zhu Q., Qu Y., Zhou X. G., Chen J. N., Luo H. R., Wu G. S. (2020). A dihydroflavonoid naringin extends the lifespan of *C. elegans* and delays the progression of aging-related diseases in PD/AD models via DAF-16. *Oxidative medicine and cellular longevity*.

[50] Jagust W. (2018). Imaging the evolution and pathophysiology of Alzheimer disease. *Nature Reviews. Neuroscience*.

[51] Wan Q. L., Fu X. D., Dai W. Y. (2020). Uric acid induces stress resistance and extends the life span through activating the stress response factor DAF-16/FOXO and SKN-1/NRF2. *Aging*.

[52] Tullet J. M. A., Hertweck M., An J. H. (2008). Direct inhibition of the longevity-promoting factor SKN-1 by insulin-like signaling in *C. elegans*. *Cell*.

[53] Ding A. J., Wu G. S., Tang B., Hong X. C., Zhu M. X., Luo H. R. (2017). Benzimidazole derivative M084 extends the lifespan of *Caenorhabditis elegans* in a DAF-16/FOXO-dependent way. *Molecular and Cellular Biochemistry*.

[54] Kaeberlein M., McVey M., Guarente L. (1999). The SIR2/3/4 complex and SIR2 alone promote longevity in Saccharomyces cerevisiae by two different mechanisms. *Genes & Development*.

[55] Zoncu R., Efeyan A., Sabatini D. M. (2011). mTOR: from growth signal integration to cancer, diabetes and ageing. *Nature Reviews. Molecular Cell Biology*.

[56] Hansen M., Flatt T., Aguilaniu H. (2013). Reproduction, fat metabolism, and life span: what is the connection?. *Cell Metabolism*.

[57] Pereira R. F., Kim E., Park Y. (2020). Cafestol increases fat oxidation and energy expenditure in *Caenorhabditis elegans* via DAF-12-dependent pathway. *Food Chemistry*.

[58] Yu L., Wang S., Chen X. (2015). Orientin alleviates cognitive deficits and oxidative stress in A*β*1-42-induced mouse model of Alzheimer's disease. *Life Sciences*.

[59] Murshid A., Eguchi T., Calderwood S. K. (2013). Stress proteins in aging and life span. *International journal of hyperthermia: the official journal of European Society for Hyperthermic Oncology, North American Hyperthermia Group*.

[60] Worley R. P. (2016). Surveillance immunity: an emerging paradigm of innate defense activation in *Caenorhabditis elegans*. *PLoS Pathogens*.

[61] Yoshida H., Okada T., Haze K. (2000). ATF6 activated by proteolysis binds in the presence of NF-Y (CBF) directly to the cis-acting element responsible for the mammalian unfolded protein response. *Molecular and Cellular Biology*.

[62] Eisermann D. J., Wenzel U., Fitzenberger E. (2016). PEK-1 is crucial for hormesis induced by inhibition of the IRE-1/XBP-1 pathway in the *Caenorhabditis elegans* mev-1 mutant. *Biochemical and Biophysical Research Communications*.

[63] Ito A., Zhao Q. C., Tanaka Y. (2021). Metolazone upregulates mitochondrial chaperones and extends lifespan in *Caenorhabditis elegans*. *Biogerontology*.

[64] Li Y. L., Ma S. C., Yang Y. T., Ye S. M., But P. P. (2002). Antiviral activities of flavonoids and organic acid from Trollius chinensis Bunge. *Journal of Ethnopharmacology*.

[65] Boominathan S. P., Sarangan G., Srikakelapu S., Rajesh S., Duraipandian C., Srikanth P. (2014). Antiviral activity of bioassay guided fractionation of Plumbago zeylanica roots against herpes simplex virus type 2. *World Journal of Pharmaceutical Sciences*.

[66] Lin Q. F., Feng S. Q., Cen Y. Z., Yang Y. T., Wang L. Y. (2004). Study on the antibacterial and antiviral activity compositions of Trollium chinensis Bunge. *Journal of Zhejiang University SCIENCE B*.

[67] Ali H., Dixit S. (2012). In vitro antimicrobial activity of flavanoids of *Ocimum sanctum* with synergistic effect of their combined form. *Asian Pacific Journal of Tropical Disease*.

[68] Nayak V., Devi P. U. (2005). Protection of mouse bone marrow against radiation-induced chromosome damage and stem cell death by the ocimum flavonoids orientin and vicenin. *Radiation Research*.

[69] Satyamitra M., Mantena S., Nair C. K. K., Chandna S., Dwarakanath B. S. (2014). The antioxidant flavonoids, orientin and vicenin enhance repair of radiation-induced damage. *Scholarena Journal of Pharmacy and Pharmacology*.

[70] Thangaraj K., Natesan K., Palani M., Vaiyapuri M. (2018). Orientin, a flavanoid, mitigates 1, 2 dimethylhydrazine-induced colorectal lesions in Wistar rats fed a high-fat diet. *Toxicology Reports*.

[71] Greer E. L., Brunet A. (2009). Different dietary restriction regimens extend lifespan by both independent and overlapping genetic pathways in *C. elegans*. *Aging Cell*.

[72] Kim J., Lee I., Seo J. (2010). Vitexin, orientin and other flavonoids from Spirodela polyrhiza inhibit adipogenesis in 3T3-L1 cells. *Phytotherapy Research*.

[73] Rosen E. D., Hsu C. H., Wang X. (2002). C/EBP*α* induces adipogenesis through PPAR*γ*: a unified pathway. *Genes & Development*.

[74] Li D., Wang Q., Yuan Z. F. (2008). Pharmacokinetics and tissue distribution study of orientin in rat by liquid chromatography. *Journal of Pharmaceutical and Biomedical Analysis*.

[75] Clemente M., Miguel M. D., Felipe K. B. (2020). Biomarkers of oxidative stress and inflammation in people with a physical disability treated with a standardized extract of Nasturtium officinale: a randomized, double-blind, and placebo-controlled trial. *Phytotherapy research: PTR*.

