# Supplementary material for: Orientin Prolongs the Longevity of Caenorhabditis elegans and Postpones the Development of Neurodegenerative Diseases via Nutrition Sensing and Cellular Protective Pathways
Source: Oxid Med Cell Longev. 2022 Feb 21;2022:8878923. doi: 10.1155/2022/8878923 (PMC8885179; doi:10.1155/2022/8878923)
Supplement: Supplementary Materials — Figure S1: graphic abstract. Table S1: mean life span of wild-type C. elegans (N2) treated with orientin in different concentrations. Table S2: effect of orientin on body bending of wild-type C. elegans (N2). Table S3: effect of orientin on lipofuscin of wild-type C. elegans (N2). Table S4: effect of orientin on resistance to high temperature (35°C), oxidation (20 mM of paraquat), and bacteria (Pseudomonas aeruginosa) in N2 worms. Table S5: effect of orientin on protein expression shown as fluorescence intensity. Table S6: effect of orientin on body bending of NL5901 ([unc-54p::α-synuclein::YFP+unc-119(+)]). Table S7: mean life span of orientin on life span of C. elegans strains. Table S8: effect of orientin on the ratio of ADP : ATP in N2 worms. Table S9: effect of orientin on mRNA expression of wild-type C. elegans (N2). Table S10: primers used for the analysis of mRNA expression levels in N2 worms. [file 8878923.f1.docx]

**Orientin Prolongs the Longevity of *Caenorhabditis elegans* and Postpones the Development of Neurodegenerative Diseases via Nutrition Sensing and Cellular Protective Pathways**

Yuan Qu^1,2^, Lin Shi^1^, Yu Liu^1^, Lv Huang^1^, Huai-Rong Luo^1,3,4*^, and Gui-Sheng Wu^5,*^

^1^ Key Laboratory for Aging and Regenerative Medicine, Department of Pharmacology School of Pharmacy, Southwest Medical University, Luzhou, Sichuan 646000, China

^2^ Department of Pharmacy Children’s Hospital of Chongqing Medical University, National Clinical Research Center for Child Health and Disorders, Ministry of Education Key Laboratory of Child Development and Disorders, Chongqing Key Laboratory of Pediatrics, Chongqing 400000, China

^3^ Central Nervous System Drug Key Laboratory of Sichuan Province, Luzhou, Sichuan 646000, China

^4^ Key Laboratory of Medical Electrophysiology, Ministry of Education & Medical Electrophysiological Key Laboratory of Sichuan, Institute of Cardiovascular Research, Southwest Medical University, Luzhou, Sichuan 646000, China

^5^ Department of Anesthesiology, Hospital (T.C.M) Affiliated to Southwest Medical University, Luzhou, Sichuan 646000, China

* Corresponding author: Drs. Huai-Rong Luo, Gui-Sheng Wu;

Phone: +86 830-3160842; Fax: +86 830-3160842.

E-mail address: [lhr@swmu.edu.cn](mailto:lhr@swmu.edu.cn), [wgs@swmu.edu.cn](mailto:wgs@swmu.edu.cn).

ORCID: <https://orcid.org/0000-0001-8912-6694>, <https://orcid.org/0000-0003-1913-9566>.

**Supplementary materials**

**Contents:**

**Figure S1. Graphic abstract.**

**Table S1. Mean lifespan of wild-type *C. elegans* (N2) treated with orientin in different concentrations.**

**Table S2. Effect of orientin on body bending of wild-type *C. elegans* (N2).**

**Table S3. Effect of orientin on lipofuscin of wild-type *C. elegans* (N2).**

**Table S4. Effect of orientin on resistance to high temperature (35 °C), oxidation (20 mM of paraquat) and bacteria (*Pseudomonas aeruginosa*) in N2 worms.**

**Table S5.** **Effect of orientin on protein expression shown as fluorescence intensity.**

**Table S6. Effect of orientin on body bending of NL5901 *([unc-54p::α-synuclein::YFP+unc-119(+)])*.**

**Table S7. Mean lifespan of orientin on lifespan of *C. elegans* strains.**

**Table S8. Effect of orientin on the ratio of ADP: ATP in N2 worms.**

**Table S9. Effect of orientin on mRNA expression of wild-type *C. elegans* (N2).**

**Table S10. Primers used for the analysis of mRNA expression levels in N2 worms.**

**Figure S1. Graphic abstract.**


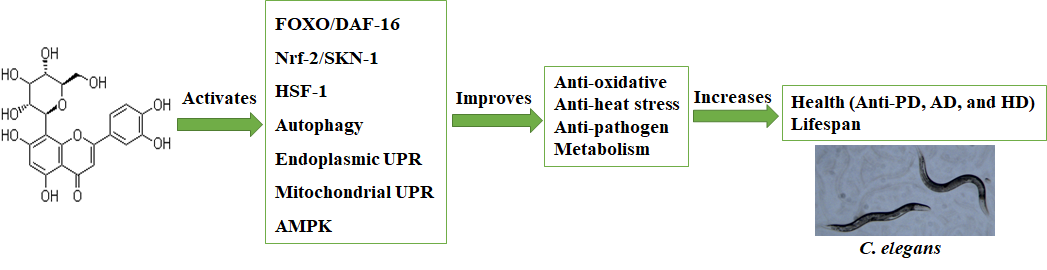


**Table S1. Mean lifespan of wild-type *C. elegans* (N2) treated with orientin in different concentrations.**

| Figure  1B and 1C Concentration |  | Control  (0 μM) | 25 μM  orientin | 50 μM  orientin | 100 μM  orientin | 200 μM  orientin |
| --- | --- | --- | --- | --- | --- | --- |
| strain |  | N2(WT) | N2(WT) | N2(WT) | N2(WT) | N2(WT) |
| Treatment |  | 20 °C/OP50  (dead) | 20 °C/OP50  (dead) | 20 °C/OP50  (dead) | 20 °C/OP50  (dead) | 20 °C/OP50  (dead) |
| Mean±SEM | EXP.1  EXP.2  EXP.3 | 20.189±0.615  20.484±0.616  20.851±0.697 | 23.411±0.600  22.533±0.874  21.933±0.696 | 24.789±0.719  24.241±0.744  23.473±0.805 | 25.070±0.806  25.028±0.832  25.704±0.810 | 24.270±0.641  23.926±0.674  25.449±0.533 |
| *P* value  VS control | EXP.1  EXP.2  EXP.3 |  | <0.001  0.004  0.192 | <0.001  <0.001  0.006 | <0.001  <0.001  <0.001 | <0.001  <0.001  <0.001 |
| N | EXP.1  EXP.2  EXP.3 | 90  95  87 | 107  60  75 | 90  79  74 | 86  72  81 | 89  95  78 |
| change in mean lifespan | EXP.1  EXP.2  EXP.3 |  | 15.959%  10.003%  5.189% | 22.784%  18.341%  12.575% | 24.176%  22.183%  23.274% | 20.213%  16.803%  22.051% |

*P*-value was analyzed by log-rank test.

N: number of dead worms.

*P* < 0.05 indicated that the experiment was statistically significant, while *p* > 0.05 indicated that the experiment was not statistically significant.

**Table S2. Effect of orientin on body bending of wild-type *C. elegans* (N2).**

| Figure | Strain | Treatment | Number of body bending Mean ± SEM | *P* value  VS  control | N | Number of body bending  Mean ± SEM | *P* value  VS  control | | N |
| --- | --- | --- | --- | --- | --- | --- | --- | --- | --- |
| 1D | N2(WT) | OP50(dead) | 5 day of adult | | | 10 day of adult | | | |
|  | EXP.1  EXP.1 | 20 °C /Control  20 °C/100 μM Orientin | 33.8±2.914  36.067±2.977 | 0.0048 | 30  30 | 24.2±1.869  29.767±2.629 | <0.001 | | 30  30 |
|  | EXP.2  EXP.2 | 20 °C /Control  20 °C/100 μM Orientin | 31.967±3.674  32.667±2.560 | 0.4034 | 30  30 | 23.8±2.428  30.833±2.478 | <0.001 | 30  30 | |
|  | EXP.3  EXP.3 | 20 °C /Control  20 °C/100 μM Orientin | 31.067±2.932  35.233±3.106 | <0.001 | 30  30 | 19.9±2.982  24.767±1.995 | <0.001 | 30  30 | |

The body movement count time was 20 seconds per nematode. N was the experimental sample size, and *p* value was calculated by two-tailed t-test. *P* < 0.05 indicated that the experiment was statistically significant.

**Table S3. Effect of orientin on lipofuscin of wild-type *C. elegans* (N2).**

| Figure | Strain | Treatments | Lipofuscin  Mean±SEM | *P* value  VS  control | Change in  mean  lipofuscin | N |
| --- | --- | --- | --- | --- | --- | --- |
| 1E | N2(WT) | OP50(dead) |  |  |  |  |
|  | EXP.1  EXP.1 | 20 °C /Control  20 °C/100 μM Orientin | 7.404±1.863  6.067±0.856 | 0.0009 | -18.062% | 30  30 |
|  | EXP.2  EXP.2 | 20 °C /Control  20 °C/100 μM Orientin | 7.399±1.237  6.014±1.096 | <0.001 | -18.713% | 30  30 |
|  | EXP.3  EXP.3 | 20 °C /Control  20 °C/100 μM Orientin | 6.942±1.474  4.750±0.581 | <0.001 | -31.574% | 26  26 |

On the 10th day of the adult worm, the accumulation of lipofuscin in intestinal tissues of the treated and untreated nematodes was photographed and counted. Fluorescence intensity was analyzed by ImageJ, and *p* value was calculated by two-tailed *t*-test, where *p* < 0.05 indicated that the experiment was statistically significant.

**Table S4. Effect of orientin on resistance to high temperature (35 °C), oxidation (20 mM of paraquat) and bacteria (*Pseudomonas aeruginosa*) in N2 worms.**

| Figure | Strain | Treatment | Mean lifespan  ±SEM | *P* value  VS  control | | change in mean lifespan | N | |
| --- | --- | --- | --- | --- | --- | --- | --- | --- |
| 2A | **N2(WT)**  EXP.1  EXP.1 | OP50(dead)  35 °C /Control  35 °C/100 μM Orientin | Hours  7.844±0.360  10.027±0.293 | <0.001 | | 27.830% | 64  73 | |
|  | EXP.2  EXP.2 | 35 °C /Control  35 °C/100 μM Orientin | 10.029±0.402  14.395±0.331 | <0.001 | | 43.534% | 70  76 | |
|  | EXP.3  EXP.3 | 35 °C /Control  35 °C/100 μM Orientin | 6.290±0.315  8.067±0.222 | <0.001 | | 28.251% | 69  90 | |
| 2C | **PS3551 *hsf-1(sy441) I*** | OP50(dead) | Hours |  |  | | |  |
|  | EXP.1  EXP.1 | 35 °C /Control  35 °C/100 μM Orientin | 5.111±0.205  5.312±0.217 | 0.434 | -3.932% | | | 90  93 |
|  | EXP.2  EXP.2 | 35 °C /Control  35 °C/100 μM Orientin | 5.984±0.197  6.126±0.187 | 0.688 | -2.372% | | | 123  127 |
|  | EXP.3  EXP.3 | 35 °C /Control  35 °C/100 μM Orientin | 4.374±0.174  4.753±0.188 | 0.125 | -8.664% | | | 91  93 |
| 2D | **PS3551 *hsf-1(sy441) I*** | OP50(dead) | Days |  |  | | |  |
|  | EXP.1  EXP.1 | 20 °C /Control  20 °C/100 μM Orientin | 19.800±0417  19.932±0.435 | 0.302 | 0.667% | | | 120  146 |
|  | EXP.2  EXP.2 | 20 °C /Control  20 °C/100 μM Orientin | 20.984±0.392  21.654±0.373 | 0.258 | 3.193% | | | 124  136 |
|  | EXP.3  EXP.3 | 20 °C /Control  20 °C/100 μM Orientin | 21.874±0.489  21.495±0.457 | 0.524 | -1.733% | | | 87  93 |
| 3A | **N2(WT)**  EXP.1  EXP.1 | Paraquat(dead)  20 °C /Control  20 °C/100 μM Orientin | Days  12.354±0.250  15.234±0.381 | <0.001 | | 23.312% | 96  77 | |
|  | EXP.2  EXP.2 | 20 °C /Control  20 °C/100 μM Orientin | 12.321±0.275  14.477±0.315 | <0.001 | | 17.498% | 106  109 | |
|  | EXP.3  EXP.3 | 20 °C /Control  20 °C/100 μM Orientin | 12.667±0.205  15.175±0.310 | <0.001 | | 19.799% | 87  80 | |
| 4A | **N2(WT)**  EXP.1  EXP.1 | PA14  20 °C /Control  20 °C/100 μM Orientin | Days  9.868±0.249  11.423±0.319 | <0.001 | | 15.758% | 76  71 | |
|  | EXP.2  EXP.2  EXP.3  EXP.3 | 20 °C /Control  20 °C/100 μM Orientin  20 °C /Control  20 °C/100 μM Orientin | 10.647±0.201  11.977±0.293  10.459±0.251  11.817±0.295 | <0.001  <0.001 | | 12.984%  12.492% | 136  129  109  131 | |
| 5D | **JIN1375 *hlh-30(tm1978) Ⅳ*** | OP50(dead) | Days |  |  | | |  |
|  | EXP.1  EXP.1 | 20 °C /Control  20 °C/100 μM Orientin | 12.710±0.368  12.333±0.419 | 0.747 | 2.966% | | | 69  66 |
|  | EXP.2  EXP.2 | 20 °C /Control  20 °C/100 μM Orientin | 11.869±0.356  11.800±0.389 | 0.765 | 0.581% | | | 61  60 |
|  | EXP.3  EXP.3 | 20 °C /Control  20 °C/100 μM Orientin | 13.917±0.396  14.684±0.339 | 0.558 | -5.512% | | | 84  79 |
|  |  |  |  |  |  | | |  |

Orientin-treated and untreated N2 nematodes were transferred into 35 °C high temperature and paraquat on the 7th day. Late L4 larvae or young adults of N2 worms were transferred on the NGM plates with *Pseudomonas aeruginosa* (PA14) at 20 °C. The survival rate was calculated.

The experimental data were processed and analyzed by SPSS26.0, represented by Kaplan-Meier survival curve. *P* values were analyzed by Log-rank test.

**Table S5. Effect of orientin on protein expression shown as fluorescence intensity.**

| Figure | | | Strain | | | Treatments | | Fluorescence  intensity  Mean±SD | | P value  VS  Control | | Change in mean | N |
| --- | --- | --- | --- | --- | --- | --- | --- | --- | --- | --- | --- | --- | --- |
| 3B | **N2(WT)** | | | OP50(dead) | | |  | |  | |  | |  |
|  | EXP.1  EXP.1  EXP.1  EXP.1 | | | 20 °C /Control  20 °C /1 mM NAC  20 °C /2 mM PQ  20 °C/100 μM Orientin | | | 4.768±1.915  3.423±0.768  13.321±1.610  4.317±1.167 | | 0.001  <0.001  0.292 | | -28.209%  179.383%  -9.458% | | 25  29  25  31 |
|  | EXP.2  EXP.2  EXP.2  EXP.2 | | | 20 °C /Control  20 °C /1 mM NAC  20 °C /2 mM PQ  20 °C/100 μM Orientin | | | 19.692±6.943  5.779±3.019  23.708±6.075  6.552±3.350 | | <0.001  0.020  <0.001 | | -70.654%  20.392%  -66.728% | | 33  45  30  43 |
|  | EXP.3  EXP.3  EXP.3  EXP.3 | | | 20 °C /Control  20 °C /1 mM NAC  20 °C /2 mM PQ  20 °C/100 μM Orientin | | | 13.726±2.551  4.740±1.751  20.893±5.250  7.924±1.404 | | <0.001  <0.001  <0.001 | | -65.470%  52.212%  -42.269% | | 29  45  29  35 |
| 3C | | **CF1553*muIs84 [(pAD76) sod-3p::GFP + rol-6(su1006)]*** | | | | OP50(dead) | |  | |  | |  |  |
|  | | EXP.1  EXP.1 | | | | 20 °C /Control  20 °C/100 μM Orientin | | 4.144±0.881  6.035±1.437 | | <0.001 | | 45.659% | 31  30 |
|  | | EXP.2  EXP.2 | | | | 20 °C /Control  20 °C/100 μM Orientin | | 4.423±0.710  6.901±1.290 | | <0.001 | | 56.039% | 37  37 |
|  | | EXP.3  EXP.3 | | | | 20 °C /Control  20 °C/100 μM Orientin | | 6.161±1.729  10.545±3.875 | | <0.001 | | 71.174% | 34  34 |
| 3D | | **LD1 *ldIs7 [skn-1B/C::GFP+pRF4 (rol-6(su1006))]*** | | | | OP50(dead) | |  | |  | |  |  |
|  | | EXP.1  EXP.1 | | | | 20 °C /Control  20 °C/100 μM Orientin | | 4.076±0.455  4.437±0.459 | | 0.002 | | 8.862% | 33  34 |
|  | | EXP.2  EXP.2 | | | | 20 °C /Control  20 °C/100 μM Orientin | | 5.950±0.903  7.636±1.320 | | <0.001 | | 28.322% | 30  30 |
|  | | EXP.3  EXP.3 | | | | 20 °C /Control  20 °C/100 μM Orientin | | 7.188±0.746  8.236±0.964 | | <0.001 | | 14.581% | 30  30 |
| 4D | | **SJ4005*zcIs4 [hsp-4::GFP] V*** | | | | OP50(dead) | |  | |  | |  |  |
|  | | EXP.1  EXP.1 | | | | 20 °C /Control  20 °C/100 μM Orientin | | 9.026±1.550  12.637±3.848 | | <0.001 | | 40% | 30  30 |
|  | | EXP.2  EXP.2 | | | | 20 °C /Control  20 °C/100 μM Orientin | | 10.557±1.011  13.356±1.930 | | <0.001 | | 26.509% | 27  27 |
|  | | EXP.3  EXP.3 | | | | 20 °C /Control  20 °C/100 μM Orientin | | 4.915±1.585  8.028±3.011 | | <0.001 | | 63.325% | 30  30 |
| 4E | | **SJ4100 *zcIs13V (hsp-6::GFP)*** | | | | OP50(dead) | |  | |  | |  |  |
|  | | EXP.1  EXP.1 | | | | 20 °C /Control  20 °C/100 μM Orientin | | 2.515±0.317  4.754±1.296 | | <0.001 | | 89.010% | 30  30 |
|  | | EXP.2  EXP.2 | | | | 20 °C /Control  20 °C/100 μM Orientin | | 4.339±0.786  6.893±1.304 | | <0.001 | | 58.872% | 30  30 |
|  | | EXP.3  EXP.3 | | | | 20 °C /Control  20 °C/100 μM Orientin | | 4.418±0.614  7.193±1.847 | | <0.001 | | 62.799% | 30  30 |
| 5A | | **BC12921 *[rCesT12G3.1::GFP + pCeh361]*** | | | | OP50(dead) | |  | |  | |  |  |
|  | | EXP.1  EXP.1 | | | | 20 °C /Control  20 °C/100 μM Orientin | | 2.971±0.573  1.792±0.358 | | <0.001 | | -39.690% | 24  24 |
|  | | EXP.2  EXP.2 | | | | 20 °C /Control  20 °C/100 μM Orientin | | 2.325±0.274  1.567±0.306 | | <0.001 | | -32.583% | 30  30 |
|  | | EXP.3  EXP.3 | | | | 20 °C /Control  20 °C/100 μM Orientin | | 2.119±0.306  1.369±0.256 | | <0.001 | | -35.39% | 24  24 |
| 6A | | **NL5901 *([unc-54p::α-synuclein::YFP+unc-119(+)])*** | | | | OP50(dead) | |  | |  | |  |  |
|  | | EXP.1  EXP.1 | | | | 20 °C /Control  20 °C/100 μM Orientin | | 24.866±8.171  18.216±7.176 | | 0.002 | | -26.744% | 30  27 |
|  | | EXP.2  EXP.2 | | | | 20 °C /Control  20 °C/100 μM Orientin | | 52.027±8.594  29.317±6.106 | | <0.001 | | -43.649% | 30  30 |
|  | | EXP.3  EXP.3 | | | | 20 °C /Control  20 °C/100 μM Orientin | | 31.575±5.381  14.549±5.896 | | <0.001 | | -53.923% | 30  28 |
| 6C | | **BZ555 *egIs1(dat-1::gfp)*** | | | | OP50(dead) | |  | |  | |  |  |
|  | | EXP.1  EXP.1  EXP.1  EXP.1 | | | | 20 °C /Control  20 °C /6-OHDA  20 °C/6-OHDA+  100 μM Orientin  20 °C /6-OHDA+  2 mM levodopa | | 24.391±3.344  6.665±1.812  14.482±2.085  17.904±2.199 | | <0.001  <0.001  <0.001 | | -72.673%  -40.626%  -26.597% | 43  31  30  42 |
|  | | EXP.2  EXP.2  EXP.2  EXP.2 | | | | 20 °C /Control  20 °C /6-OHDA  20 °C/6-OHDA+  100 μM Orientin  20 °C /6-OHDA+  2 mM levodopa | | 19.759±1.902  9.028±1.991  12.438±1.344  14.682±1.885 | | <0.001  <0.001  <0.001 | | -54.311%  -37.052%  -25.697% | 20  35  25  24 |
|  | | EXP.3  EXP.3  EXP.3  EXP.3 | | | | 20 °C /Control  20 °C /6-OHDA  20 °C/6-OHDA+  100 μM Orientin  20 °C /6-OHDA+  2 mM levodopa | | 26.793±2.846  6.301±1.520  14.953±1.952  20.398±1.874 | | <0.001  <0.001  <0.001 | | -76.485%  -44.190%  -23.869% | 37  34  39  43 |
| 6E | | **AM141 *(rmIs133)[unc-54p:: Q40::YFP]*** | | | OP50(dead) | | |  | |  | |  |  |
|  | | EXP.1  EXP.1 | | | | 20 °C /Control  20 °C/100 μM Orientin | | 52.281±7.225  42.281±4.237 | | <0.001 | | -19.127 | 32  32 |
|  | | EXP.2  EXP.2 | | | | 20 °C /Control  20 °C/100 μM Orientin | | 42.966±5.353  38.828±4.060 | | 0.002 | | -9.631 | 29  29 |
|  | | EXP.3  EXP.3 | | | | 20 °C /Control  20 °C/100 μM Orientin | | 49.6±4.733  39.92±7.121 | | <0.001 | | -13.593 | 25  25 |

*P* value was determined by comparison between the control group and the experimental group in each independent experiment. The data in the experimental group were counted as the number of normal dead nematodes. The experimental data were processed and analyzed by SPSS26.0 with Kaplan-Meier survival curve. *P* value was obtained by log-rank test analysis.

N: number of dead nematodes.

**Table S6. Effect of orientin on body bending of NL5901 *([unc-54p::α-synuclein::YFP+unc-119(+)])*.**

| Figure | | Strain | | Treatment | Number of body bending Mean ± SEM | | P value  VS  control | N | | Number of body bending  Mean ± SEM | P value  VS  control | | N |
| --- | --- | --- | --- | --- | --- | --- | --- | --- | --- | --- | --- | --- | --- |
| 6B | **NL5901** | | OP50(dead) | | 5 day of adult | | | | 10 day of adult | | | | |
|  | EXP.1  EXP.1 | | 20 °C /Control  20 °C/100 μM Orientin | | 32.533±2.589  35.367±3.261 | 0.0005 | | 30  30 | 20.9±4.069  29.367±3.516 | | <0.001 | | 30  30 |
|  | EXP.2  EXP.2 | | 20 °C /Control  20 °C/100 μM Orientin | | 18.367±2.927  21.8±3.885 | 0.0003 | | 30  30 | 9.1±2.357  12.567±2.753 | | <0.001 | 30  30 | |
|  | EXP.3  EXP.3 | | 20 °C /Control  20 °C/100 μM Orientin | | 17.633±4.468  17.3±4.034 | 0.0767 | | 30  30 | 12.133±3.019  15.767±3.019 | | <0.001 | 30  30 | |

The body movement count time was 20 seconds per nematode. N was the experimental sample size, and *P* value was calculated by two-tailed t-test. *P* < 0.05 indicated that the experiment was statistically significant.

The body movement count time was 20 seconds per nematode. N was the experimental sample size, and *P* value was calculated by two-tailed t-test. *P* < 0.05 indicated that the experiment was statistically significant.

**Table S7. Mean lifespan of orientin on lifespan of *C. elegans* strains.**

| Figure | Strain | Treatment | Mean lifespan  ±SEM | P value  VS  control | change in mean lifespan | N |
| --- | --- | --- | --- | --- | --- | --- |

| 3E | | **EU1**  ***skn-1(zu67) IV*** | | OP50(dead) | Days |  | |  | |  | |  |  |
| --- | --- | --- | --- | --- | --- | --- | --- | --- | --- | --- | --- | --- | --- |
|  | | EXP.1  EXP.1 | | 20 °C /Control  20 °C/100 μM Orientin | 16.830±0.441  17.296±0.449 | 0.569 | | 2.769% | | 88  81 | |  |  |
|  | | EXP.2  EXP.2 | | 20 °C /Control  20 °C/100 μM Orientin | 18.089±0.373  17.419±0.312 | 0.177 | | -3.704% | | 112  118 | |  |  |
|  | | EXP.3  EXP.3 | | 20 °C /Control  20 °C/100 μM Orientin | 18.195±0.511  18.077±0.571 | 0.869 | | -0.648% | | 77  65 | |  |  |
| 5C | | **VC893**  [***atg-18***](https://cgc.umn.edu/strain/search?st1=atg-18&sf1=all)***(***[***gk378***](https://cgc.umn.edu/strain/search?st1=gk378&sf1=all)***) V*** | | OP50(dead) | | Days | |  | |  | |  | |
|  | | EXP.1  EXP.1 | | 20 °C /Control  20 °C/100 μM Orientin | | 14.686±0.425  15.016±0.418 | | 0.744 | | 2.247% | | 70  64 | |
|  | | EXP.2  EXP.2 | | 20 °C /Control  20 °C/100 μM Orientin | | 14.700±0.390  14.367±0.494 | | 0.961 | | -2.265% | | 70  60 | |
|  | | EXP.3  EXP.3 | | 20 °C /Control  20 °C/100 μM Orientin | | 15.770±0.407  16.017±0.495 | | 0.485 | | 1.566% | | 74  60 | |

| 6D | **CL4176 *dvIs27 [myo-3p::A-Beta (1-42)::let-851 3'UTR) + rol-6(su1006)] X*** | OP50(dead) | Hours |  |  | |  |
| --- | --- | --- | --- | --- | --- | --- | --- |
|  | EXP.1  EXP.1 | 20 °C /Control  20 °C/100 μM Orientin | 7.275±0.322  9.500±0.429 | <0.001 | | 30.584% | 102  96 |
|  | EXP.2  EXP.2 | 20 °C /Control  20 °C/100 μM Orientin | 7.065±0.385  10.000±0.403 | <0.001 | 41.543% | | 77  98 |
|  | EXP.3  EXP.3 | 20 °C /Control  20 °C/100 μM Orientin | 7.495±0.510  9.514±0.723 | 0.006 | 26.938% | | 91  70 |

| 7A | **CB1370 *daf-2(e1370) III*** | | OP50(dead) | | Days | | | |  | | | | |  | |  | | |  |  |
| --- | --- | --- | --- | --- | --- | --- | --- | --- | --- | --- | --- | --- | --- | --- | --- | --- | --- | --- | --- | --- |
|  | EXP.1  EXP.1 | | 20 °C /Control  20 °C/100 μM Orientin | | 44.273±1.411  44.605±1.158 | | | | 0.499 | | | | | 0.749% | | 88  86 | | |  |  |
|  | EXP.2  EXP.2 | | 20 °C /Control  20 °C/100 μM Orientin | | 42.135±1.072  43.310±1.024 | | | | 0.416 | | | | | 2.789% | | 96  100 | | |  |  |
|  | EXP.3  EXP.3 | | 20 °C /Control  20 °C/100 μM Orientin | | 42.956±1.240  42.578±1.024 | | | | 0.429 | | | | | -0.879% | | 90  109 | | |  |  |
| 7B | **TJ1052 *age-1(hx546) II*** | | OP50(dead) | | Days | | | |  | | | | |  | |  | | |  |  |
|  | EXP.1  EXP.1 | | 20 °C /Control  20 °C/100 μM Orientin | | 24.104±0.753  24.262±1.163 | | | | 0.278 | | | | | 0.655% | | 77  61 | | |  |  |
|  | EXP.2  EXP.2 | | 20 °C /Control  20 °C/100 μM Orientin | | 23.423±0.977  23.397±1.172 | | | | 0.687 | | | | | -0.111% | | 78  73 | | |  |  |
|  | EXP.3  EXP.3 | | 20 °C /Control  20 °C/100 μM Orientin | | 24.233±0.748  24.333±0.833 | | | | 0.751 | | | | | 0.412% | | 103  93 | | |  |  |
| 7C | **RB759 *akt-1(ok525) V*** | | OP50(dead) | | Days | | | |  | | | | |  | |  | | |  |  |
|  | EXP.1  EXP.1 | | 20 °C /Control  20 °C/100 μM Orientin | | 25.872±0.448  26.229±0.428 | | | | 0.932 | | | | | 1.380% | | 78  70 | | |  |  |
|  | EXP.2  EXP.2 | | 20 °C /Control  20 °C/100 μM Orientin | | 25.395±0.478  24.652±0.389 | | | | 0.041 | | | | | -2.926% | | 86  92 | | |  |  |
|  | EXP.3  EXP.3 | | 20 °C /Control  20 °C/100 μM Orientin | | 21.980±0.632  21.204±0.634 | | | | 0.374 | | | | | -3.530% | | 99  93 | | |  |  |
| 7D | **VC204 *akt-2(ok393) X*** | | OP50(dead) | | Days | | | |  | | | | |  | |  | | |  |  |
|  | EXP.1  EXP.1 | | 20 °C /Control  20 °C/100 μM Orientin | | 22.242±0.778  22.426±0.671 | | | | 0.812 | | | | | 0.827% | | 62  68 | | |  |  |
|  | EXP.2  EXP.2 | | 20 °C /Control  20 °C/100 μM Orientin | | 22.685±0.661  23.114±0.638 | | | | 0.857 | | | | | 1.891% | | 73  70 | | |  |  |
|  | EXP.3  EXP.3 | | 20 °C /Control  20 °C/100 μM Orientin | | 22.469±0.847  22.792±0.776 | | | | 0.947 | | | | | 1.437% | | 64  72 | | |  |  |
| 7E | **CF1038 *daf-16(mu86) I*** | | OP50(dead) | | Days | | | |  | | | | |  | |  | | |  |  |
|  | EXP.1  EXP.1 | | 20 °C /Control  20 °C/100 μM Orientin | | 16.612±0.314  17.289±0.221 | | | | 0.965 | | | | | 4.075% | | 139  142 | | |  |  |
|  | EXP.2  EXP.2 | | 20 °C /Control  20 °C/100 μM Orientin | | 17.804±0.361  17.805±0.379 | | | | 0.506 | | | | | 0.005% | | 56  82 | | |  |  |
|  | | EXP.3  EXP.3 | 20 °C /Control  20 °C/100 μM Orientin | | | 19.702±0.514  20.000±0.620 | | | | 0.547 | | | | 1.512% | | 94  63 | | |  |  |
| 8A | **DA1116 *eat-2(ad1116) II*** | | OP50(dead) | | Days | | | |  | | | | |  | |  | | |  |  |
|  | EXP.1  EXP.1 | | 20 °C /Control  20 °C/100 μM Orientin | | 34.825±0.627  36.548±0.616 | | | | 0.061 | | | | | 4.947% | | 126  124 | | |  |  |
|  | EXP.2  EXP.2 | | 20 °C /Control  20 °C/100 μM Orientin | | 33.491±0.637  34.881±0.697 | | | | 0.077 | | | | | 4.150% | | 112  118 | | |  |  |
|  | EXP.3  EXP.3 | | 20 °C /Control  20 °C/100 μM Orientin | | 33.216±0.637  34.047±0.726 | | | | 0.166 | | | | | 2.502% | | 116  127 | | |  |  |
| 8C | **RB754 *aak-2(ok524) X*** | | OP50(dead) | | Days | | | |  | | | | |  | |  | | |  |  |
|  | EXP.1  EXP.1 | | 20 °C /Control  20 °C/100 μM Orientin | | 18.733±0.416  18.750±0.393 | | | | 0.918 | | | | | 0.091% | | 60  60 | | |  |  |
|  | EXP.2  EXP.2 | | 20 °C /Control  20 °C/100 μM Orientin | | 18.50±0.413  18.286±0.360 | | | | 0.437 | | | | | -1.157% | | 60  63 | | |  |  |
|  | EXP.3  EXP.3 | | 20 °C /Control  20 °C/100 μM Orientin | | 18.786±0.332  18.381±0.337 | | | | 0.450 | | | | | -2.156% | | 84  84 | | |  |  |
| 8D | **VC199 *sir-2.1(ok434) IV*** | | OP50(dead) | | Days | | | |  | | | | |  | |  | | |  |  |
|  | EXP.1  EXP.1 | | 20 °C /Control  20 °C/100 μM Orientin | | 18.282±0.431  18.707±0.439 | | | | 0.450 | | | | | 2.325% | | 71  75 | | |  |  |
|  | EXP.2  EXP.2 | | 20 °C /Control  20 °C/100 μM Orientin | | 17.698±0.428  18.013±0.480 | | | | 0.567 | | | | | 1.780% | | 86  76 | | |  |  |
|  | EXP.3  EXP.3 | | 20 °C /Control  20 °C/100 μM Orientin | | 18.647±0.420  18.860±0.463 | | | | 0.317 | | | | | 1.142% | | 102  100 | | |  |  |
| 8E | **VC1027**  [***daf-15***](https://cgc.umn.edu/strain/search?st1=daf-15&sf1=all)***(***[***ok1412***](https://cgc.umn.edu/strain/search?st1=ok1412&sf1=all)***)/***[***nT1***](https://cgc.umn.edu/strain/search?st1=nT1&sf1=all)***IV; +/***[***nT1***](https://cgc.umn.edu/strain/search?st1=nT1&sf1=all)***V)*** | | OP50(dead) | | Days | | | |  | | | | |  | |  | | |  |  |
|  | EXP.1  EXP.1 | | 20 °C /Control  20 °C/100 μM Orientin | | 17.022±0.395  19.549±0.375 | | | | <0.001 | | | | | 14.845% | | 90  133 | | |  |  |
|  | EXP.2  EXP.2 | | 20 °C /Control  20 °C/100 μM Orientin | | 17.064±0.369  18.629±0.344 | | | | 0.006 | | | | | 9.171% | | 94  116 | | |  |  |
|  | EXP.3  EXP.3 | | 20 °C /Control  20 °C/100 μM Orientin | | 20.077±0.454  21.493±0.452 | | | | 0.003 | | | | | 7.053% | | 104  134 | | |  |  |
| 9A | | **CB4876 *clk-1(e2519) III*** | | OP50(death) | Days | | |  | | | | |  | | | |  | | |  |
|  | | EXP.1  EXP.1 | | 20 °C /Control  20 °C/100 μM Orientin | 28.545±0.721  29.549±0.583 | | | 0.981 | | | | | 3.52% | | | | | 66  71 | | |
|  | | EXP.2  EXP.2 | | 20 °C /Control  20 °C/100 μM Orientin | 29.989±0.502  29.632±0.604 | | | 0.830 | | | | | -1.19% | | | | 89  87 | | |  |
|  | | EXP.3  EXP.3 | | 20 °C /Control  20 °C/100 μM Orientin | | 29.869±0.784  29.721±0.764 | | | | | | 0.937 | -0.50% | | | | 61  61 | | |  |
| 9B | | **MQ887 *isp-1(qm150) IV*** | | OP50(death) | | Days | | | |  | | | | |  | |  | | |  |
|  | | EXP.1  EXP.1 | | 20 °C /Control  20 °C/100 μM Orientin | | 24.536±0.432  23.907±0.377 | | | | 0.981 | | | | | -2.56% | | 97  118 | | |  |
|  | | EXP.2  EXP.2 | | 20 °C /Control  20 °C/100 μM Orientin | | 24.985±0.609  24.304±0.539 | | | | 0.443 | | | | | -2.73% | | 68  79 | | |  |
|  | | EXP.3  EXP.3 | | 20 °C /Control  20 °C/100 μM Orientin | | 24.216±0.480  23.312±0.470 | | | | 0.124 | | | | | -3.73% | | 88  93 | | |  |
| 9C | | **TK22 *mev-1 (kn1) III*** | | OP50(death) | | Days | | | |  | | | | |  | |  | | |  |
|  | | EXP.1  EXP.1 | | 20 °C /Control  20 °C/100 μM Orientin | | 14.115±0.250  14.056±0.262 | | | | 0.889 | | | | | -0.42% | | 87  90 | | |  |
|  | | EXP.2  EXP.2 | | 20 °C /Control  20 °C/100 μM Orientin | | 15.442±0.246  15.352±0.242 | | | | 0.776 | | | | | -0.58% | | 86  88 | | |  |
|  | | EXP.3  EXP.3 | | 20 °C /Control  20 °C/100 μM Orientin | | 15.143±0.249  15.118±0.213 | | | | 0.904 | | | | | -0.17% | | 63  76 | | |  |
| 9D | | **CF1903**  ***glp-1(e2141) III*** | | OP50(dead) | | Days | | |  | | | | |  | |  | | |  |  |
|  | | EXP.1  EXP.1 | | 20 °C /Control  20 °C/100 μM Orientin | | 22.627±0.989  22.348±1.017 | | | 0.83 | | | | | -1.233% | | 67  66 | | |  |  |
|  | | EXP.2  EXP.2 | | 20 °C /Control  20 °C/100 μM Orientin | | 22.246±1.027  21.957±0.952 | | | 0.675 | | | | | -1.299% | | 65  70 | | |  |  |
|  | | EXP.3  EXP.3 | | 20 °C /Control  20 °C/100 μM Orientin | | 20.475±0.983  20.575±0.975 | | | 0.710 | | | | | 0.488% | | 61  73 | | |  |  |
| 9E | | **AA89**  ***daf-12 (rh274) X*** | | OP50(dead) | | Days | | |  | | | | |  | |  | | |  |  |
|  | | EXP.1  EXP.1 | | 20 °C /Control  20 °C/100 μM Orientin | | 25.726±0.833  26.016±0.816 | | | 0.898 | | | | | 1.127% | | 62  64 | | |  |  |
|  | | EXP.2  EXP.2 | | 20 °C /Control  20 °C/100 μM Orientin | | 22.229±0.669  22.831±0.612 | | | 0.867 | | | | | 2.708% | | 70  65 | | |  |  |
|  | | EXP.3  EXP.3 | | 20 °C /Control  20 °C/100 μM Orientin | | 21.517±0.671  21.267±0.856 | | | 0.481 | | | | | -1.162% | | 60  60 | | |  |  |

*p*-value was analyzed by log-rank test.

N: number of dead worms.

*P* <0.05 indicated that the experiment was statistically significant, while *P* > 0.05 indicated that the experiment was not statistically significant.

**Table S8. Effect of orientin on the ratio of ADP: ATP in N2 worms.**

| Fig. | Strain | Treatments | ATP  (mg  /kg) | ADP  (mg  /kg) | AMP  (mg  /kg) | ADP:ATP | Sample size  (g) |
| --- | --- | --- | --- | --- | --- | --- | --- |
| 8B | N2(WT) | OP50(dead) |  |  |  |  |  |
|  | EXP.1  EXP.1 | 20 °C /Control  20 °C/100 μM Orientin | 222.6  116.5 | 227.7  220.3 | 226.0  106.9 | 1.023  1.891 | 1  1 |
|  | EXP.2  EXP.2 | 20 °C /Control  20 °C/100 μM Orientin | 226.5  118.1 | 241.9  220.5 | 226.3  104.1 | 1.068  1.867 | 1  1 |
|  | EXP.3  EXP.3 | 20 °C /Control  20 °C/100 μM Orientin | 226.3  114.1 | 234.1  228.5 | 233.5  103.1 | 1.034  2.002 | 1  1 |

**Table S9. Effect of orientin on mRNA expression of wild-type *C. elegans* (N2).**

| Figure | Gene | EXP.1 | EXP.2 | EXP.3 | Mean±SD |
| --- | --- | --- | --- | --- | --- |
|  | *Control* | 1 | 1 | 1 | 1 |
| 2B | *hsf-1* | 1.664583 | 1.675973 | 1.624267 | 0.02716785 |
|  | *hsp-12.6* | 4.645252 | 4.583295 | 4.636836 | 0.033606071 |
|  | *hsp-16.1* | 1.376694 | 1.647669 | 1.448915 | 0.140324786 |
|  | *hsp-16.2* | 3.560002 | 3.603384 | 3.557859 | 0.025687598 |
|  | *hsp-6* | 1.604777 | 1.569481 | 1.676772 | 0.054681457 |
|  | *hsp-60* | 1.132988 | 1.198993 | 1.243803 | 0.055744054 |
| 4B | *F55G11.4* | 1.345772 | 1.279858 | 1.312401 | 0.032957867 |
|  | *irg-1* | 4.576857 | 4.416162 | 4.902752 | 0.247924808 |
| 4C | *pgp-8* | 1.675069612 | 1.670444026 | 1.721401664 | 0.028180182 |
|  | *ire-1* | 1.890355479 | 1.817036977 | 1.92283896 | 0.054198461 |
|  | *xbp-1* | 1.555050153 | 1.857352611 | 1.467179514 | 0.204671717 |
|  | *ubl-5* | 1.312065023 | 1.425614673 | 1.363326363 | 0.056863992 |
|  | *pek-1* | 1.951776595 | 1.882249546 | 2.015758293 | 0.066773565 |
|  | *atf-6* | 1.854313975 | 3.764716425 | 3.279567467 | 0.993005081 |
|  | *atfs-1* | 2.082574598 | 2.136306973 | 2.059634594 | 0.039353241 |
|  | *aak-2* | 4.658203728 | 4.630526284 | 4.759818712 | 0.068078604 |
|  | *sir-2.1* | 1.87752 | 1.632393 | 2.132038 | 0.249837208 |
| 5B | *bec-1* | 2.151049 | 1.96001 | 2.360708 | 0.200421091 |
|  | *lgg-1* | 3.797642 | 3.986254 | 3.617953 | 0.184168514 |
| 7G | *daf-16* | 1.508237 | 1.496607 | 1.445305 | 0.033485579 |
|  | *ctl-1* | 1.666991 | 1.676392 | 1.708388 | 0.021701986 |
|  | *ctl-3* | 1.561788 | 1.338383 | 1.667121 | 0.167865809 |
|  | *sod-3* | 4.738993 | 3.369201 | 3.489093 | 0.758612135 |
|  | *dod-3* | 2.693989 | 2.195508 | 2.967625 | 0.391476344 |
| 8F | *fat-1* | 1.383277828 | 1.328619743 | 1.255990792 | 0.063854601 |
|  | *fat-3* | 1.648418646 | 1.603054123 | 1.728863893 | 0.063714828 |
|  | *fat-6* | 3.792410899 | 3.967977456 | 3.293888959 | 0.349700596 |
|  | *acs-2* | 2.346471 | 3.336412 | 2.779277 | 0.496270142 |
|  | *lipl-4* | 3.370342 | 4.128468 | 5.057126 | 0.84482727 |

**Table S10. Primers used for the analysis of mRNA expression levels in N2 worms.**

| Gene | Type | Sequence |
| --- | --- | --- |
| *cdc-42* | Forward primer  Reverse primer | CTGCTGGACAGGAAGATTACG  CTCGGACATTCTCGAATGAAG |
| *hsf-1* | Forward primer  Reverse primer | TTGACGACGACAAGCTTCCAGT  AAAGCTTGCACCAGAATCATCCC |
| *hsp-12.6* | Forward primer  Reverse primer | GTGATGGCTGACGAAGGAAC  GGGAGGAAGTTATGGGCTTC |
| *hsp-16.1* | Forward primer  Reverse primer | GTCACTTTACCACTATTTCCGTCCAGCTCAACGTTC  CAACGGGCGCTTGCTGAATTGGAATAGATCTTC |
| *hsp-16.2* | Forward primer  Reverse primer | CTGCAGAATCTCTCCATCTGAGTC  AGATTCGAAGCAACTGCACC |
| *hsp-6* | Forward primer  Reverse primer | AGGAACAACAGAGTAAGATTTTC  TCGATTTGGTCCTTGGAAAG |
| *hsp-60* | Forward primer  Reverse primer | AGGAGAAGCTTAATGAGCG  ACACGGTCCTTCTTCTCT |
| *dve-1* | Forward primer  Reverse primer | TCGAGGCCTCATACAAGAA  AAGAGGTTTTCCACAGTGTC |
| *F55G11.4* | Forward primer  Reverse primer | GGATCCGTGTATTTGGCTGGAATCG  GTGAAGACATATGTGCTCCCGCGTT |
| *irg-1* | Forward primer  Reverse primer | AAGCAGCATGCGTATTTTCA  GCAGCTTCTCCTTTTTCTCC |
| *ire-1* | Forward primer  Reverse primer | TTACATCACGCTTCCCTCGG  CGAGCACGAACATCATCGGA |
| *xbp-1* | Forward primer  Reverse primer | ACGTATTTATGTGCTCCCAG  TATCATCGCCAAGAAGTTGT |
| *ubl-5* | Forward primer  Reverse primer | ACTGGAACACGATGGGAAAAGA  AGCTCGAAATTGAATCCCTCGT |
| *pek-1* | Forward primer  Reverse primer | GGGACTAGTCGCAACAGAGC  GAAGGAAATCCCGCGACTCT |
| *atf-6* | Forward primer  Reverse primer | GATCTACTGAAGACCGGAAACA  AAGGCAGAAGCACGTAGTCT |
| *atfs-1* | Forward primer  Reverse primer | GAAGGTAACCGCACCGATCA  GCTGGTCGTTCTATGCCTCA |
| *daf-16* | Forward primer  Reverse primer | CCAGACGGAAGGCTTAAAACT  ATTCGCATGAAACGAGAATG |
| *ctl-1* | Forward primer  Reverse primer | GAATGTGAAGAATTATTTCGCTGA  AACTCGATTCCTGGGACGAT |
| *ctl-2* | Forward primer  Reverse primer | CAAGGAACTACTTCGCTGAGG  AATGAGTGTCGGTGTACGAGAA |
| *ctl-3* | Forward primer  Reverse primer | AGTAAATCTTCAAAATGCCAATG  GGTGGGGTTCCTGATTTCTAT |
| *sod-3* | Forward primer  Reverse primer | AGCATCATGCCACCTACGTGA  CACCACCATTGAATTTCAGCG |
| *dod-3* | Forward primer  Reverse primer | AAGCCATGTTCCCGAATGAG  GCTGCGAAAAGCAAGAAAATG |
| *daf-9* | Forward primer  Reverse primer | GAGGGCATTCTCCGCAAGT  CCACTGCTGAAGTCGAAATCC |
| *skn-1* | Forward primer  Reverse primer | AGTGTCGGCGTTCCAGATTTC  GTCGACGAATCTTGCGAATCA |
| *gst-4* | Forward primer  Reverse primer | TCCGTCAATTCACTTCTTCCG  AAGAAATCATCACGGGCTGG |
| *bec-1* | Forward primer  Reverse primer | AGCATCCGTTGAGGTTGGATT  TTCGAAGAGCGTCAGAGCAAT |
| *lgg-1* | Forward primer  Reverse primer | AACAACTTTGAGAAGCGTCGTGCC  TCTTCTGGACGAAGTTGGATGCGT |
| *fat-6* | Forward primer  Reverse primer | GCTCACTATTTCGGATGGA  GATGGAAGTTGTGACCTCC |
| *fard-1* | Forward primer  Reverse primer | GGGTTTTTGGGAAAGGTGAT  CCACCGATTGCTTTCAATTT |
| *acs-2* | Forward primer  Reverse primer | TCAATCCTCGTATCCCGCCG  TATTCGAGGTTCCGTGGATG |
| *lipl-4* | Forward primer  Reverse primer | ATGGCCGAGAAGTTCCTACATCGT  GGTGAATTGGCGACCCAATCGAAA |
| *lips-17* | Forward primer  Reverse primer | ATCTGTTGCTGGAGCCAATCG  TATCCAACTTTATCGTCTCC |
